# Cross-Study Meta-Analysis of Blood Transcriptomes in Type 2 Diabetes

**DOI:** 10.3390/ijms262412046

**Published:** 2025-12-15

**Authors:** Aleksandr A. Tkachenko, Ziravard N. Tonyan, Yulia A. Nasykhova, Yury A. Barbitoff, Iaroslav N. Renev, Maria M. Danilova, Anastasiia A. Basipova, Olga B. Glavnova, Dmitrii E. Polev, Sergey V. Chepanov, Sergey A. Selkov, Nikita V. Golovkin, Margarita E. Vlasova, Andrey S. Glotov

**Affiliations:** 1 D.O. Ott Research Institute of Obstetrics, Gynecology and Reproductology, Mendeleevskaya Line 3, 199034 Saint Petersburg, Russiaelenamariamassa@gmail.com (M.M.D.); anamikhajlova@gmail.com (A.A.B.);; 2 St. Martyr George City Hospital, 194354 Saint Petersburg, Russia

**Keywords:** RNAseq, type 2 diabetes, blood transcriptome, meta-analysis, RNA sequencing, expression profiling

## Abstract

Type 2 diabetes (T2D) is a chronic metabolic disorder with an estimated prevalence of over 422 million individuals affected globally. Since the advent of genomics, numerous studies have been conducted to elucidate T2D pathogenetic mechanisms and define genetic loci affecting T2D susceptibility. Transcriptomic studies, including bulk and single-cell RNA sequencing, play an important role both in discerning molecular mechanisms of the disease and in identifying potential T2D biomarkers. In this study, we performed bulk RNA-seq of whole blood of nine T2D patients and nine control subjects and performed meta-analysis of these data with seven publicly available blood RNA-seq datasets of T2D patients. Our analysis showed that the changes in the gene expression between different studies show very low concordance; moreover, a substantial number of differentially expressed genes (DEGs) was identified in only three out of eight datasets, with only five DEGs—*FBLN2*, *TPCN1*, *PC*, *SHANK1*, and *PLD4*—identified in all three of those datasets. Nevertheless, cross-study meta-analysis identified a broad set of 2065 DEGs, including 713 genes that have not been identified in any of the source studies. These genes showed a significant enrichment of GO terms indicating neutrophil activation and proliferation and included several genes that have not been implicated in type 2 diabetes previously. Taken together, our study highlights challenges associated with biomarker discovery from blood transcriptomics in T2D and suggests novel genes that may be considered as such biomarkers.

## 1. Introduction

Type 2 diabetes (T2D) is a chronic metabolic disorder characterized by insulin resistance and relative insulin deficiency. It is a significant public health concern worldwide, with an estimated prevalence of over 422 million individuals affected globally [1]. This number is projected to rise, making diabetes one of the leading causes of morbidity and mortality [2]. It is associated with numerous severe microvascular and macrovascular complications, including retinopathy, nephropathy, cardiovascular diseases, and neuropathy, which substantially impact patients’ quality of life and healthcare systems [3].

The global prevalence of T2D necessitates a deeper understanding of its underlying mechanisms to develop more effective prevention and treatment strategies. Recent advancements in transcriptomics and single-cell technologies have revolutionized our ability to investigate the cellular and molecular landscapes of T2D. Transcriptomic analysis provides insights into gene expression patterns in T2D, revealing pathways involved in insulin signaling, metabolic regulation, and inflammation. The importance of inflammation driven by immune cells in the pathogenesis of T2D has been repeatedly demonstrated [4,5]. This makes research focused on the analysis of gene expression in whole blood cells especially relevant. Transcriptomic markers of T2D have been investigated in studies utilizing expression arrays and RNA sequencing (RNA-seq). While expression arrays allow for the analysis of a gene set limited by the design of microarrays, RNA-seq allows investigating the entire transcriptome. A study focused on the analysis of transcriptomic markers of T2D in whole blood using RNA-seq demonstrated differential expression of genes associated with inflammation, insulin resistance, and mitochondrial dysfunction [6]. However, the variability in experimental design, sample processing, and analytical methods across individual RNA-seq studies poses significant challenges in deriving robust biological insights. Cross-study meta-analysis of RNA-seq data enables researchers to harmonize findings from diverse datasets, allowing for the identification of consistent molecular signatures and pathways underlying disease mechanisms. By integrating transcriptomic data from multiple independent studies, we can enhance statistical power, mitigate batch effects, and uncover reproducible biomarkers of disease progression.

In this study, we aimed to perform a comprehensive meta-analysis of publicly available RNA-seq datasets from T2D patients and healthy controls in order to validate key transcriptomic alterations, analyze discrepancies across studies, and highlight the importance of cross-study integration in advancing our understanding of T2D pathogenesis.

## 2. Results

### 2.1. Differential Expression Analysis Across Cohorts

**Figure 1:**
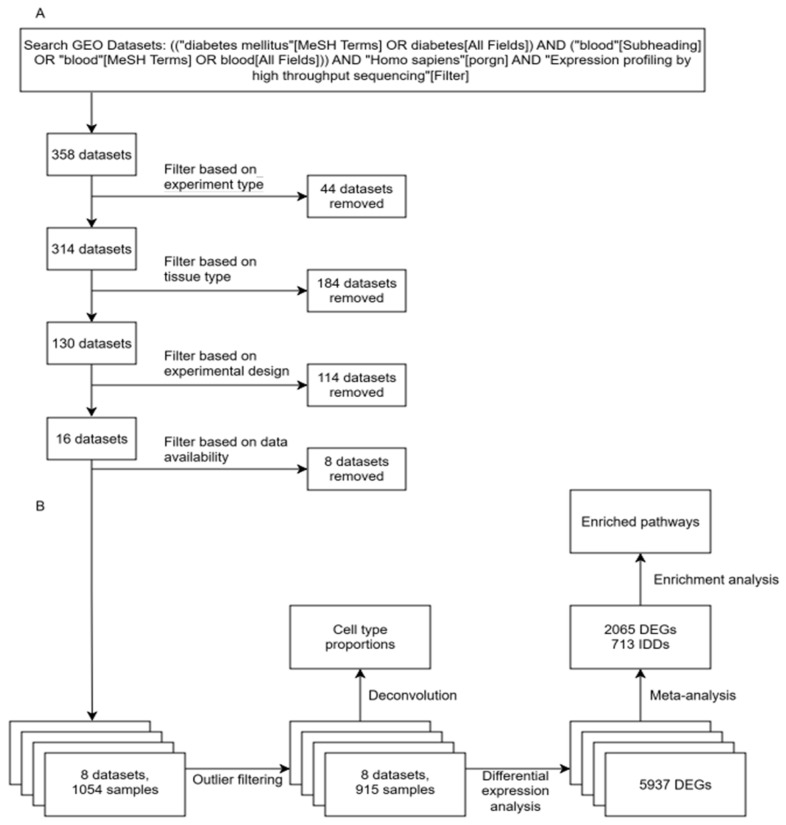
Flow chart of data analysis in the current study. (**A**) Search and selection strategy for datasets. (**B**) Data analysis process. IDD—integration-driven discovery.

For meta-analysis of blood transcriptomes in type 2 diabetes, we conducted a search across datasets in Gene Expression Omnibus (GEO). Search term and selection process are represented in Figure 1A. The details of the selection process are provided in the Section 4, but, in broad terms, we aimed to select bulk RNA sequencing datasets that allow the comparison of blood transcriptomes between healthy and T2D-affected individuals. We analyzed bulk RNA sequencing data for samples in eight datasets (including one first presented in the current study). Information about datasets is summarized in Table 1. A total of 1054 initial samples were reduced to 915 samples after the outlier filtering procedure described in Methods. Out of eight analyzed datasets, six were representing whole blood samples, while two others were peripheral blood mononuclear cells (PBMCs). The overall data processing scheme is shown in Figure 1B.

Each dataset featured a unique perspective on studying the biology of type 2 diabetes, which was reflected in different study designs. In order to make comparison between cohorts possible, we utilized the dream (differential expression for repeated measures) analysis of differential expression as implemented in the variancePartition package, which allowed us to analyze cohorts with or without repeated measurements. Designs used for every study to fit linear models and contrasts to obtain fold change estimates and *p*-values are presented in Table 2. In all cases, in addition to a tested coefficient associated with diabetes status (condition), we included biological sex (either author-supplied annotation or inferred from the data) and some other descriptors that might explain a sufficient portion of variation in the experiment.

**Figure 2:**
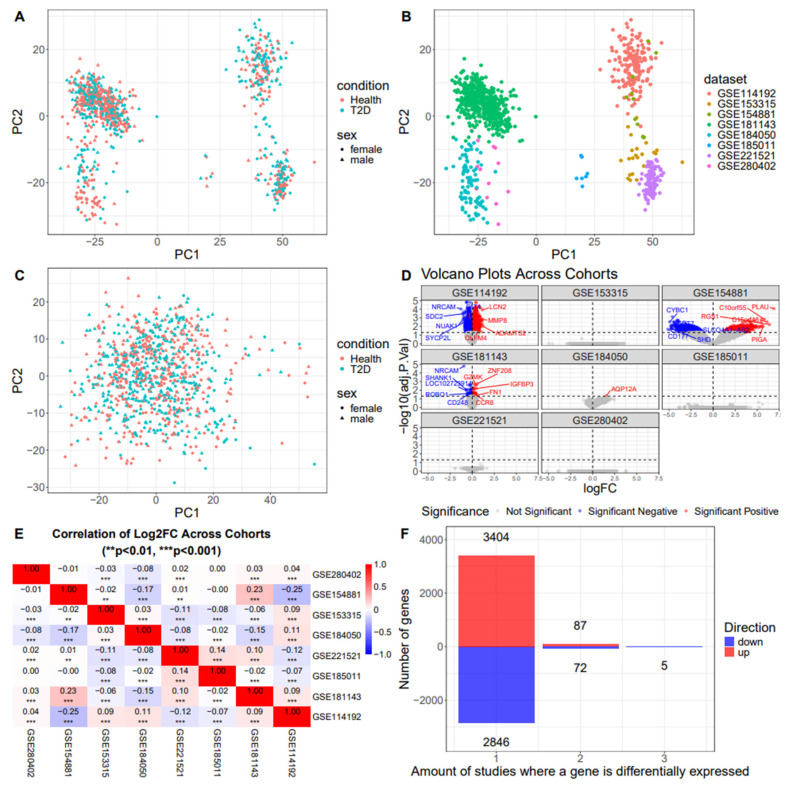
Differential expression analysis results to individual datasets. (**A**) PC biplot of all analyzed samples colored based on the diabetes status and sex of patients. (**B**) PC biplot of all analyzed samples colored based on the dataset. (**C**) PC biplot of all analyzed samples after batch correction for dataset; color of dots indicates diabetes status and shape corresponds to sex. (**D**) Volcano plots for individual datasets; x-axis represents logFC, y—negative log of adjusted *p*-values. (**E**) Pairwise correlations of effect sizes (logFoldChange estimates) in different datasets. (**F**) Number of differentially expressed genes (DEGs) that share direction of expression change between studies; x-axis shows the amount of studies that share a DEG; y-axis—amount of genes in each category and the direction of change.

Before running differential expression analysis, we performed principal component analysis (PCA) of the combined dataset. We found that the samples clustered into several distinct groups; however, no clear separation between healthy controls and T2D patients was observed (Figure 2A). At the same time, samples were clearly separated by dataset, showing a pronounced batch effect in gene expression measurements (Figure 2B). Correction for this effect, however, did not improve the separation of cases and controls (Figure 2C), indicating high levels of inter-individual variability in gene expression.

Out of 39,176 genes included in the study, effect sizes of diabetes status were significant for 5937 genes in at least one study analyzed (with a Benjamini–Hochberg FDR threshold of 0.05). Results tables for individual studies are presented in Supplemental Table S1. MA-plots, PCA biplots, and variance partition plots for all studies are presented in Figures S1–S8. The number of differentially expressed genes (DEGs) ranged from 0 (in four out of eight studies) to 3417 unique genes in different datasets (Table 3, Figure 2D). Notably, results from different studies were not strongly correlated, as evident from correlation heatmap on Figure 2E. Lack of correlation between studies with regard to expression changes between T2D samples and healthy controls is further evidenced by the amount of DEGs shared between studies (Figure 2F). The majority of genes are not differentially expressed in any dataset, and there are no DEGs that are shared between more than three studies. Only five DEGs share direction in three studies—*FBLN2*, *TPCN1*, *PC*, *SHANK1*, and *PLD4*. Normalized expression values for these genes in all samples are shown in Supplementary Figure S9.

Changes in RNA expression levels in blood samples associated with type 2 diabetes might be linked to the cell composition of the samples. To explore this possible connection, we ran the quanTIseq deconvolution algorithm as implemented in the immunedeconv package on TPM-normalized expression estimates for all samples to predict proportions of cell types for every sample. Aggregated information for median cell proportions in all datasets is represented in Supplementary Figure S10. In the majority of samples and datasets, the uncharacterized cell type represents the highest proportion, although for certain cohorts, neutrophils are the most abundant cell type. After fitting a linear model that accounts for dataset, sex, and diabetes status, only B cell proportion shows a significant difference between T2D and healthy samples (BH-adjusted *p*-value—0.047), albeit with a very small effect size (−0.0054 log fold change between T2D and healthy samples). Importantly, the most pronounced changes in cell type composition between control individuals and T2D patients were observed in datasets with no or very few DEG (Supplementary Figure S11), suggesting that changes in cell type composition do not significantly skew the results of differential expression analysis.

### 2.2. Meta-Analysis of Differential Expression Analysis Results

**Figure 3:**
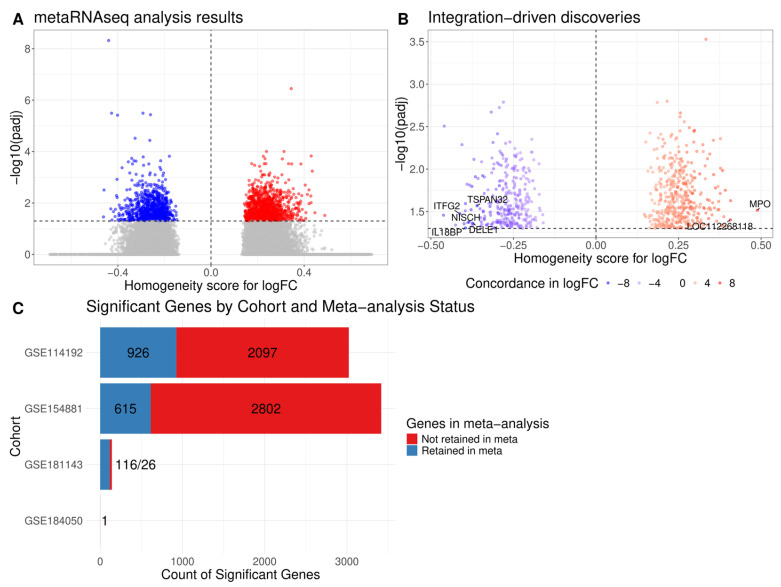
Meta-analysis results and integration-driven discoveries. (**A**) Meta-analysis results for all genes in the study; x-axis depicts the custom homogeneity score, and y-axis depicts negative log of adjusted *p*-values from meta-analysis. Blue and red dots are discoveries with negative and positive homogeneity scores for logFC, respectively. (**B**) Same picture as in A, but only for integration-driven discoveries. Color corresponds to concordance scores for logFCs. (**C**) Histogram of DEGs from original studies that were retained as discoveries after meta-analysis.

After analyzing each dataset, we turned our attention to the meta-analysis of individual results using the metaRNAseq package. Combination of raw *p*-values was performed with the inverse normal method. For combination of fold changes, we created a custom score that ranges from −1 to 1 and penalizes discordance in both signs and absolute values of the individual logFoldChanges. A large negative value of this score means that logFoldChanges are mostly concordant and downregulated and a large positive score means concordant upregulation. The discordant logFoldChanges result in scores close to 0. Our meta-analysis resulted in 2065 genes deemed differentially expressed after the combination of all the datasets (Figure 3A). Among them, 713 genes were integration-driven discoveries (IDDs) that were discovered as DEGs exclusively in the meta-analysis and were not differentially expressed in any of the initial datasets (Figure 3B), which corresponds to an integration-driven discovery rate of 34.53%. The full list of IDD DEGs is presented in Supplemental Table S2. Only seven genes had the same direction of effect for comparison of type 2 diabetes samples and healthy samples in all eight datasets: *LOC112268118* and *MPO* had positive logFC, and *IL18BP*, *DELE1*, *TSPAN32*, *ITFG2*, and *NISCH* all had negative logFC. The majority of genes that were declared discoveries in original studies were not retained after meta-analysis, amounting to an overall integration-driven revision rate of 77.22% (Figure 3C).

### 2.3. Pathway Enrichment Analysis

**Figure 4:**
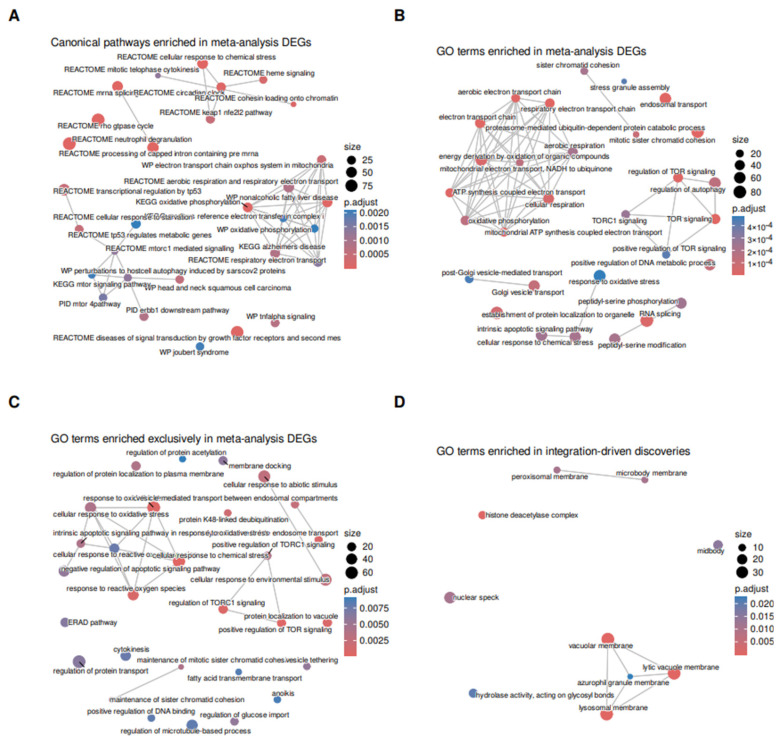
Enrichment analysis for meta-analysis results. (**A**) Canonical pathways enrichment for meta-analysis discoveries (**B**) GO term enrichment for meta-analysis discoveries. (**C**) GO terms enriched for meta-analysis discoveries but not for any of the initial datasets. (**D**) GO term enrichment for meta-analysis integration-driven discoveries. On all subplots, dots are connected based on pairwise similarity of GO terms, with size and color of dots representing number of DEGs in the pathway and adjusted *p*-value, respectively. Only top 30 categories in terms of enrichment are shown for subplots (**A**–**C**).

To analyze patterns in meta-analysis discoveries and, especially, in integration-driven discoveries, we performed enrichment analysis of these groups of genes, using Gene Ontology (GO) terms and canonical pathways (CPs) and Hallmark (H) gene sets from the Molecular Signatures Database (MSigDB) as groups being tested for overrepresentation in our results. Meta-analysis DEGs were enriched in 467 GO categories (296 in Biological Process (BP) ontology, 58—in Molecular Function (MF), and 113 in Cellular Component (CC)), 232 canonical pathways, and 4 Hallmark pathways. Enrichment map plots for most enriched pathways in GO, CP, and Hallmarks are shown in Figure 4A,B and Figure S12A,B. In both GO- and CP-based analysis, several groups of related terms and pathways were identified. These include genes involved in neutrophil degranulation, p53-mediated transcriptional regulation, signaling by growth factor receptors and tumor necrosis factor alpha (TNFa) (Figure 4A). Additionally, enrichment analysis revealed over-representation of genes connected to oxidative phosphorylation and electron transport chain, components of the mTOR signaling pathway, and genes involved in cell division and chromatin organization (Figure 4B).

Additionally, we compared GO enrichment in individual datasets with enrichment in meta-analysis DEGs and found that 135 GO terms (103 in BP, 15 in MF, and 17 in CC) are only enriched in meta-analysis results but not in any of the initial datasets (Figure 4C). These included several notable groups of important biological processes, such as response to oxidative stress and apoptotic signaling, components of vesicular transport, mTOR signaling, and endoplasmic reticulum associated degradation (ERAD) pathway. The biological relevance of these groups of genes will be considered in detail in Discussion. For IDD genes, only 10 GO terms (0 in BP, 1 in MF, 9 in CC) were significantly enriched (Figure 4D). These included one notable group of several interconnected cellular component terms (vacuolar membrane, lytic vacuole membrane, azurophil granule membrane) that is likely connected to the effector functions on neutrophils (see Discussion).

## 3. Discussion

In this study, we have aggregated blood RNA sequencing data from eight independent studies to identify systematic gene expression differences between T2D patients and controls. Our results show that the detection of DEGs between T2D and control subjects is challenging; however, meta-analysis of the results allowed us to identify novel pathways and genes in blood cells that appear to be associated with T2D status and may serve as potential biomarkers for the disease.

Our meta-analysis includes gene expression datasets derived from peripheral blood mononuclear cells (PBMCs) in addition to those from whole blood. While whole blood contains all circulating cells, PBMCs represent a critical fraction comprising lymphocytes (T cells, B cells, NK cells) and monocytes. These cell types are central players in the immune and inflammatory pathways that are highly relevant to the pathophysiology of type 2 diabetes [9]. Inclusion of both types of studies allows us to identify robust transcriptional changes that are not likely to be artifacts of a particular sample type.

In the present work, we have aggregated blood RNA sequencing data from eight independent studies of varying sizes to identify systematic gene expression differences between T2D patients and controls. The integration of datasets with disparate sample sizes is a recognized approach in meta-analyses, but it necessitates methodological adjustments to ensure validity. To this end, we employed a meta-analysis approach that explicitly weighs each study’s contribution based on its sample size. This mitigates potential biases from size discrepancies and addresses concerns such as the “small study effect,” which is further alleviated in our case as study inclusion was not based on prior differential expression results. Our results show that the detection of DEGs between T2D and control subjects is challenging on an individual-study level; however, the meta-analysis of the results allowed us to identify novel pathways and genes in blood cells that appear to be consistently associated with T2D status and may serve as potential biomarkers for the disease.

The first major finding of our study is the low number of DEGs detected in the majority of the studies that were analyzed, as well as the extremely low overlap between sets of DEGs identified in different studies. In this study, we used a technique to remove potential outliers that relies on concordance between several outlier detection methods and has been shown to increase reliability of downstream data analysis [10]. Despite removing outlier samples, correlation between effect sizes for T2D status in different datasets was very low (Figure 2A). These results can be explained by a variety of factors that affect the observed expression levels of genes in whole blood-based transcriptomics. Such factors may include (but are not limited to) (i) variation in gene expression levels between ethnic groups and between individuals in the same group, (ii) low power to detect significant gene expression differences due to insufficient sample size or presence of additional confounders, and (iii) technical variation in gene expression measurements associated with methodological aspects of blood-based transcriptomics.

Our analysis highlights that inter-individual variation is a prominent feature of blood transcriptomic studies. This is evidenced by the substantial unexplained variance in gene expression observed across all datasets (Figures S1–S8), consistent with previous reports of wide variation in whole blood, including our earlier study of expression changes associated with exercise [11]. A key contributor to this heterogeneity is the variation in blood cell type proportions, which differs both between individuals and between studies (Figures S10 and S11). This suggests that cell type-specific analyses, such as single-cell RNA sequencing, may offer greater resolution for detecting nuanced expression changes, as demonstrated in prior T2D research [9]. However, modeling cell type-specific gene expression changes in bulk RNA-seq data is challenging. In addition to blood cell proportions, ongoing immune response to pathogens may also affect gene expression profiles of individual donors in a profound manner [12].

When technical factors are considered, abundance of globin gene transcripts appears to be an important factor that can add noise to gene expression measurements. Globin depletion has been shown to increase concordance between technical replicates that might improve discovery of true biological variation [13]. It was also demonstrated that bioinformatic depletion of globins performs worse than kit-based depletion prior to sequencing [14]. However, none of the studies in our meta-analysis report using the globin depletion methods, and, indeed, our analysis shows that globin genes (including *HBA1*, *HBA2*, and *HBB*) and ribosomal RNA genes are among the top five most expressed genes in the vast majority of samples in our analysis, and their total expression levels vary substantially between samples (Figure S13). Besides globin depletion, the protocols used for library preparation and sequencing also differ between studies. This factor may be one of the most important forces driving large-scale variation in gene expression profiles between studies irrespective of T2D status (Figure 2D). Batch correction for dataset largely mitigates this problem (Figure 2E), and in the case of the present study, this between-dataset variation was handled by analyzing each dataset separately and combining individual results.

Despite the inherent heterogeneity in individual transcriptomic studies, our integrative analysis successfully identified a core set of reproducible gene expression changes in T2D, both when comparing sets of DEGs identified in individual studies and using meta-analysis approach. In the former case, we detected five genes that were differentially expressed and concordant in direction in all three studies in which a substantial number of DEGs were identified. These include *FBLN2*, *TPCN1*, *PC*, *SHANK1*, and *PLD4*.

The most promising of the detected genes is *PC*. The *PC* gene encodes pyruvate carboxylase, an enzyme that plays a crucial role in the early stages of gluconeogenesis by converting pyruvate into oxaloacetate in the mitochondria [15]. Reduced expression of this gene has been observed in β-cells and islets from diabetic patients and mice [16,17,18,19,20]. Additionally, pyruvate carboxylase has been shown to stabilize the MDM2 protein, thereby limiting p53-dependent senescence in β-cells [19,20]. Increased expression of *PC* in the INS-1 cell line leads to enhanced insulin secretion and cell proliferation [21]. Conversely, suppression of *PC* results in decreased insulin expression in insulinoma cells [22]. Following pancreatic resection in rats, an increase in the activity of the pyruvate carboxylase pathway is noted, which is associated with accelerated β-cells proliferation [23]. The PC-urea cycle pathway suppresses nitric oxide (NO) synthesis, thereby protecting β-cells from inflammatory cytotoxicity [24]. Another mechanism of PC-mediated protection of β-cells involves glutathione synthesis and enhanced antioxidant activity to counteract inflammation [25].

The *FBLN2* gene encodes fibulin-2, a glycoprotein secreted into the extracellular matrix that is responsible for tissue growth, development, and remodeling [26]. This gene has been associated with Goldenhar syndrome [27], human breast cancer [28], and non-small cell lung cancer [29]. Currently, there are no research findings that demonstrate a clear relationship between the expression of this gene and the risk of T2D; however, there is evidence implicating fibulin-2 in adipogenesis processes. For instance, a multi-omics study showed increased expression of *FBLN2* in high-fat diet obesity-resistant mice [30]. Furthermore, research by Fujimoto et al. indicated elevated expression of this gene in insulin-resistant diabetic mice, which the authors attributed to potential changes in adipocyte morphology associated with increased obesity risk during insulin resistance development [31,32].

The *TPCN1* gene encodes two-pore channel protein 1, an ion channel located in lysosomes and endosomes that is responsible for ion transport [33]. Research studies conducted on mice suggest a potential involvement of *TPCN1* in the insulin secretion process. A proposed mechanism involves the release of calcium from acidic stores through TPC in response to NAADP, which is synthesized in β-cells upon glucose elevation, leading to membrane depolarization associated with insulin secretion [34]. Notably, functional knockout of two-pore channel protein 2 (*TPCN2*) in pancreatic β-cells does not affect glucose tolerance, as its function is fully compensated by increased expression of *TPCN1*. In contrast, complete knockout of *TPCN1* results in significant impairment of glucose tolerance and glucose-stimulated insulin secretion [35]. Furthermore, this gene has been associated with obesity, which may be a risk factor for T2D. For instance, a study conducted on male mice found that knockout of *TPCN1* and *TPCN2* leads to obesity in adulthood due to impaired lipid availability for thermogenesis in brown adipose tissue [36]. Additionally, an epigenome-wide association study conducted by Orozco et al. reported that altered methylation of the *TPCN1* gene is associated with obesity [37]. Mice with complete knockout of the *TPCN1* gene also exhibited a tendency for increased fat tissue accumulation [38].

*SHANK1* is a gene that encodes a scaffold protein localized in the postsynaptic density of glutamatergic synapses [39]. It is well-established that mutations in this gene are associated with human neurodevelopmental disorders [40,41]. Concordant downregulation of *SHANK1* in three studies might hint at the link between T2D and neurological complications or impaired neuronal regulation of metabolism. Currently, it has been demonstrated that the Shank protein directly interacts with the insulin receptor substrate protein p53 [42,43]. A double knockout of Shank1 and Shank2 in mice has shown a significant reduction in the activation levels of the Akt signaling pathway [44], which is one of the primary signaling pathways involved in the pathogenesis of T2D and associated obesity [45].

*PLD4* encodes phospholipase D4, which is associated with various pathologies, including Alzheimer’s disease, rheumatoid arthritis, systemic sclerosis, and other autoimmune and autoinflammatory diseases [46,47]. The active involvement of this gene in inflammatory regulation processes may be one reason for the altered expression observed in T2D, a condition where immune inflammation plays a significant role in its pathogenesis [48]. In a model of human obesity using pigs, the influence of this gene on fat deposition has been demonstrated. Additionally, its effect on phospholipase activity in mice has also been associated with adipose tissue accumulation [49].

Meta-analysis allowed us to identify a rich set of 713 DEGs, which have not been observed in any of the source studies. Most notably, this group of integration-driven DEGs is enriched in several pathways that could all be explained in the context of increased immune response, especially neutrophil response. Azurophile granules are a telltale sign of neutrophils, and other enriched categories corroborate neutrophil involvement: lytic vacuole and vacuolar membrane term enrichment also point at the main function of neutrophils as phagocytic cells. Additionally, neutrophils generate a respiratory burst as a way to eliminate pathogens, which explains enrichment in the peroxisome membrane pathway. Enrichment in nuclear speck and midbody could be interpreted as signs of active transcription and cell proliferation, respectively, which might point at active immune response. The role of neutrophils in type 1 and type 2 diabetes is widely acknowledged (reviewed in [50]), and our results support these findings, even though we were not able to detect differences in predicted neutrophil proportions between T2D samples and healthy controls when controlling for sex and dataset.

Integration-driven discoveries also include other genes that have been implicated in the pathogenesis of diabetes. Notably, several genes from the ERAD (endoplasmic-reticulum-associated protein degradation) pathway stand out among the IDD DEGs—*EDEM3*, *GET4*, *JKAMP*, *MAN1C1*, *RCN3*, *RHBDD2*, and *SELENOS*. The ERAD pathway has been implicated in the pathophysiology of diabetes in a variety of ways. First, it has been shown that ERAD is essential for proper insulin secretion by pancreatic beta cells, supporting degradation of misfolded pro-insulin [51] and preventing loss of beta-cell identity [52]. Second, ERAD has been shown to contribute to T2D outside of pancreatic cell function. For example, ERAD has been shown to regulate leptin signaling in neurons by affecting the localization of leptin receptors [53]. Another mechanism of ERAD effects is the modulation of insulin signaling pathway upon ER stress. Dysfunctional ERAD may lead to insulin resistance in insulin target tissues [54]. However, no study has previously demonstrated changes in activity of the ERAD pathway and the associated proteins in blood cells. Hence, our results may indicate that the phenomenon of ER stress in T2D patients may be far more ubiquitous and fundamental than previously anticipated.

Some of the IDD genes from the ERAD pathway have also been previously described as potentially involved in diabetes and its complications. For example, *EDEM3* was shown to be involved in diabetic kidney disease in mice [55], and knockout of its regulator, *miR-379*, alleviates negative impact of high fat diet on kidney alterations in mice [56]. *GET4* and *JKAMP* have recently been identified as valuable biomarkers in diabetic cardiomyopathy in type 1 diabetes [57]. Variations in RCN3 were shown to be associated with the blood level of fructosamine—an important biomarker for diabetes [58]. Taken together, these data support the relevance of the identified ERAD-related DEGs. It is important to emphasize, however, that the majority of the newly discovered DEGs related to ERAD have not been previously implicated in T2D, making them possible targets for further research.

Besides pathways enriched in IDD genes, several pathways were identified as enriched for meta-analysis DEGs but were not found in any of the source studies. Perhaps the most notable of these pathways is the Mammalian Target of Rapamycin (mTOR) signaling pathway (Figure 4B). mTOR is a crucial regulator of cell death and proliferation that responds to a variety of stimuli, with glucose being one of the most important of these stimuli. Evidence supporting the role of mTOR in T2D is abundant (reviewed in [59]); however, it is important to note that a significant overrepresentation of mTOR complex 1 (mTORC1) signaling pathway components could only be identified after meta-analysis. This might be explained by low fold change in expression of these genes (as well as genes involved in ERAD and ROS response) in T2D patients compared to controls. Despite this fact, the basic role played by mTOR, as well as by ERAD, in T2D suggests that these genes may nevertheless be considered as potential reliable biomarkers of T2D.

A very prominent cluster of semantically similar enriched meta-specific pathways corresponded to chemical and oxidative stress (Figure 4B). Several genes from that drive the enrichment on these pathways have been discussed in the literature for the relevance in the context of T2D. A recent study has discovered an upregulation of *PRDX1* gene encoding an antioxidant and redox sensor in T2D cells [60]. *PRKAA1* encodes a subunit of AMPK (AMP-activated protein kinase), a key energy sensor. AMPK activation improves insulin sensitivity and suppresses gluconeogenesis, making it a central player in T2D metabolism [61]. *IL-18BP* encodes IL-18 binding protein, which regulates the pro-inflammatory cytokine IL-18. IL-18 is implicated in chronic inflammation, insulin resistance, and the development of T2D. IL-18BP acts as a natural inhibitor of this pathway. The levels of both IL-18 and IL-18BP were shown to be changed in type 2 diabetes in a recent study [62]. The *RHOB* gene encoding Ras homolog gene family, member B protein, and *SELENOS* have recently been identified as biomarkers for diabetic retinopathy [63,64].

The meta-analysis results are also enriched in the RNA splicing category. The enrichment of this pathway is driven by several genes that have a direct connection to diabetes. *PIK3R1* encodes the regulatory subunit (p85α) of PI3-Kinase, a central signaling molecule in the insulin signaling pathway. When insulin binds its receptor, it activates PI3K, which is the first step in triggering glucose uptake. Mutations in *PIK3R1* are directly associated with insulin resistance and T2D [65]. *SLC38A2* encodes a sodium-coupled neutral amino acid transporter (SNAT2), which transports glutamine and other amino acids into β-cells [66]. *SLC38A2* is upregulated in β cells in diabetes as a response to the translational repression associated with ER stress [67]. Another important driver of RNA splicing enrichment directly relevant to diabetes, *ERN1* (*IRE1α*), is also connected to ER stress. This is a central sensor of endoplasmic reticulum (ER) stress. In T2D, high nutrient demand leads to ER stress in beta-cells and insulin-responsive tissues, triggering the Unfolded Protein Response (UPR). *IRE1α* is a key arm of the UPR, and its sustained activation can lead to beta-cell apoptosis and insulin resistance [68]. It is also worth noting that dysregulation of RNA splicing is widely discussed as a crucial mechanism of type 2 diabetes. A recent study showed that glucose-induced alternative splicing of *MEF2D* is connected to the chronic inflammation in type 2 diabetes [69]. Another recent article discusses the disruption of splicing in beta-cells in the context of T2D [70]. The authors discover a novel *HNF1A-A1CF* transcription-splicing axis regulating splicing in beta-cells that is suppressed in β cells from T2D individuals.

Some integration-driven discoveries are interesting in the context of type 2 diabetes individually. One such gene detected only in meta-analysis is *GREM2*. This gene encodes gremlin 2, a protein that plays a crucial role in organ growth and tissue differentiation during embryonic development [71]. It was shown that fasting levels of circulating Grem2 are significantly lower in patients with type 2 diabetes compared to a healthy control group [72]. Furthermore, according to several studies, Grem2 is associated with the progression of diabetic nephropathy caused by podocyte apoptosis [72,73,74]. It is also noteworthy that Grem2 is one of the key regulators of adipocyte differentiation and adipogenesis [75]. The level of circulating Grem2 has been observed in patients with central obesity and visceral adiposity, which are known risk factors for type 2 diabetes [76,77].

Several genes from the IDD group might explain immune system involvement in diabetes comorbidities. *LY75* and *FOXRED2* may be associated with diabetic vascular aging through their involvement in immune damage to the vascular microenvironment [78]. The expression of the *LY75* gene, responsible for the synthesis of lymphocyte antigen 75, is characterized by resistance to obesity in diabetes-prone mice [79]. *FOXRED2*, which encodes FAD-dependent oxidoreductase domain-containing 2, is also involved in ERAD. Its expression has been shown to change in alpha, beta, and PP islet cells under oxidative stress induced by a high-fat, high-fructose diet in rats [80]. *NEK7*, encoding NIMA-related kinase 7, is a crucial factor involved in chronic immune inflammation in type 2 diabetes by triggering the activation of the *NLRP3* inflammasome complex. *NLRP3* is well known to be responsible for the chronic inflammation triggered by hyperglycemia [81]. Increased expression of the *NEK7* gene has been reported in patients with gestational diabetes [82], and suppression of *NLRP3* inflammasome activation related to *NEK7* overexpression has been shown to mitigate diabetes-induced muscle atrophy [83].

Another integration-driven discovery, *CPNE3*, which encodes a calcium-dependent membrane-binding protein, has also been described in patients with diabetes. Its expression is inversely correlated with the level of glycated hemoglobin and is reduced in islet cells of patients with hyperglycemia. A study conducted in 2021 demonstrated that silencing of Cpne3 in a rat insulinoma cell line suppresses glucose-stimulated insulin secretion [84].

The differentially expressed *C1QTNF12* (*CTRP12*, adipolin) is of interest as one of the regulators of insulin sensitivity, acting by enhancing insulin signaling in the liver and adipose tissue. Additionally, adipolin suppresses gluconeogenesis and promotes glucose uptake by acting directly through the PI3K-Akt signaling pathway [85].

The methylenetetrahydrofolate reductase (*MTHFR*) gene, which was found in DEGs in meta-analysis, is also well described in the context of predisposition to type 2 diabetes. It is known that certain variants of this gene are associated with hyperhomocysteinemia, which leads to vascular damage, one of the key causes of diabetes-related mortality and disability [86]. The polymorphic variant C677T in the *MTHFR* gene is associated with an increased risk of developing type 2 diabetes in the Asian population [87], and the combination of the polymorphic variant 1298AA with hypermethylation of *MTHFR* has been observed in patients with hyperglycemia and elevated levels of total cholesterol and LDL cholesterol [88].

Additionally, it is interesting to note the *ZNF496* gene, which, according to single-cell sequencing data, is one of the main regulators that promote diabetic nephropathy [89]. Another DEG detected in this study, *PM20D2*, may serve as a marker for type 2 diabetes in women with obesity, as they exhibit reduced expression of this gene in diabetes [90]. The DEG *PALD1* is a negative regulator of insulin signaling in American Indians and is associated with type 2 diabetes in this population [91]. *KAT5*, encoding lysine acetyltransferase 5, is involved in the pathogenesis of diabetic retinopathy by modulating autophagy processes through epigenetic regulation of autophagy-related gene 7 [92].

## 4. Materials and Methods

### 4.1. Study Cohorts and Participants

Peripheral blood samples were collected from 8 healthy volunteers and 8 unrelated individuals with T2D. T2D was diagnosed based on the World Health Organization criteria. The inclusion criteria for the control group were age over 30 years and no history of diabetes. Patients with type 1 diabetes, gestational diabetes, newly diagnosed diabetes mellitus (less than 1 year), acute or decompensated liver and kidney disease, autoimmune disorders, malignancies, and under 30 years of age were excluded from the study. The levels of fasting blood glucose (FBG), high-density lipoprotein (HDL), low-density lipoprotein (LDL), total cholesterol, and creatinine were measured in a fasting blood sample. Body height and body weight were measured, and the body mass index (BMI) was calculated in all the participants. The clinical and biochemical parameters of all the participants enrolled in the study are summarized in Table 3. The control group was selected to closely match the T2D group in order to minimize differences and exclude the influence of the factors listed in Table 3 on gene expression. When comparing the control group and the T2D group, the most pronounced differences were detected in fasting glucose levels, which were higher in T2D patients (*p*-value = 0.0096). Differences were also observed in BMI and WHR, both of which were elevated in the T2D group (*p*-values = 0.0007 and 0.01, respectively). These differences are likely associated with abdominal obesity and hyperglycemia, both of which are components of metabolic syndrome. Additionally, although there were no statistically significant differences in LDL levels between the two groups, it is noteworthy that LDL levels were elevated in both groups, which may partly be attributed to the lack of strict dietary restrictions imposed on the study participants. Nevertheless, the absence of differences in this parameter may indicate that it does not influence the experimental outcomes or the identification of DEGs when comparing the groups. Furthermore, no comorbidities associated with elevated LDL were documented in the control group.

Written informed consent for the research was obtained from all the recruited patients and healthy donors. The study was conducted in accordance with the Declaration of Helsinki and approved by the Institutional Ethics Committee of D.O. Ott Institute of Obstetrics, Gynecology, and Reproductology (protocol #130 from 16 July 2020). This study was performed using large-scale research facilities #3076082 “Human Reproductive Health”.

### 4.2. Blood Sample Collection

Whole blood samples were collected from the study subjects in 2020 in anticoagulant Improvacuter K2EDTA 9 mL tubes (Guangzhou Improve Medical Instruments Co., Ltd., Guangzhou, China) after an overnight fasting period of 12 h. The blood samples (n = 16) for the bulk RNA sequencing experiment were then immediately aliquoted in RNAse-free cryotubes without centrifugation (Fluidx Ltd., Cheshire, UK) and stored at −80 °C until use (in 2024) to prevent freeze–thaw cycles, according to ISBER recommendations for repositories [93].

### 4.3. Total RNA Isolation

Whole blood samples were retrieved from cryostorage and thawed at room temperature. The total RNA was isolated from 300 μL of whole blood using a TRIzol reagent (Invitrogen, Carlsbad, CA, USA) with chloroform, purificated with PureLink Total RNA Blood Kit (Invitrogen™, Carlsbad, CA, USA) following the manufacturer’s instructions and eluted with 45 μL RNase-free water. Total RNA was tested for purity using the NanoDrop 2000 spectrophotometer (Thermo Fisher Scientific, Waltham, MA, USA) and quantified with the Qubit™ RNA High Sensitivity Assay Kit and Qubit 2.0 Fluorometer (both Invitrogen™, Carlsbad, CA, USA).

### 4.4. RNA-Seq Library Preparation

Libraries were constructed from 150 ng of previously extracted total RNA using the TruSeq Stranded Total RNA library preparation kit (Illumina, Inc., San Diego, CA, USA), according to the manufacturer’s protocol. Briefly, after ribosomal RNA depletion and RNA fragmentation, first and second strand cDNA were synthesized, and 3’ ends were adenylated, followed by adapter ligation and enrichment of DNA fragments. The library’s size distribution was analyzed with Agilent 2200 TapeStation and Agilent High Sensitivity D1000 ScreenTape (both Agilent Technologies, Inc., Waltham, MA, USA).

### 4.5. Sequencing

Indexed libraries were pooled at equimolar ratios. RNA-seq was conducted identically for both experiments on Illumina HiSeq 2500 System, with 75 bp paired-end reads using HiSeq Rapid SBS Kit (all Illumina, Inc., San Diego, CA, USA).

### 4.6. Gene Expression Omnibus Datasets

Search for datasets eligible for the meta-analysis was performed on Gene Expression Omnibus among GEO Datasets using the following search terms: ((“diabetes mellitus” [MeSH Terms] OR diabetes [All Fields]) AND (“blood” [Subheading] OR “blood” [MeSH Terms] OR blood [All Fields])) AND “Homo sapiens” [porgn] AND “Expression profiling by high throughput sequencing” [Filter] in August 2025. Results were then manually filtered for relevance according to the following inclusion criteria: type of RNA sequencing should be only bulk RNA-seq; biological samples should only include either whole blood or PBMC; experimental design should allow comparison between type 2 diabetes patients and healthy controls while controlling for other factors.

Gene expression data from other studies was downloaded from GEO in the form of NCBI-generated (processing procedure is described in [94]) raw counts and TPM-normalized values. Data processing procedure for that includes mapping of reads to genome assembly GCA_000001405.15 using HISAT2. Runs that pass a 50% alignment rate are further processed with Subread featureCounts, which outputs a raw count file for each run.

### 4.7. Bulk RNA-Seq Data Analysis

In accordance with the procedure used for data processing in GEO, RNA sequencing data obtained in this study was aligned to genome assembly GCA_000001405.15 using HISAT2 v. 2.2.1 and counted with Subread featureCounts v. 2.1.1.

To remove spurious sources of variation in RNA sequencing experiments, a comprehensive outlier detection approach based on [9] was used. It combines three complementary methods: hierarchical clustering analysis (HCA), robust sparse principal component analysis (robpca function in the package rospca v. 1.1.1 [95]), and robust PCA using the PcaGrid algorithm. Thresholds for filtering outliers were set as follows: cophenetic correlation coefficient threshold for HCA was set to 0.8, maximum silhouette coefficient threshold for HCA—0.25, and outlier probability thresholds for both robust PCA approaches—0.975. A sample was declared an outlier if at least two methods out of three agree.

For samples without author-supplied sex annotation, we inferred biological sex directly from the count data by comparing counts of Y-chromosome encoded gene *KDM5D* and *XIST* based on their salience as tissue-independent sex markers [96]. Analysis of the donors for whom sex information was provided by the authors of original studies confirmed near-perfect accuracy of such inference (Supplementary Figure S14).

Differential expression was analyzed in a dataset-wise manner using the variancePartition package v. 1.34.0 [97]. Enrichment of pathways in differential expression data was calculated with clusterProfiler v. 4.12.6 [98].

Combination of *p*-values from individual studies using the inverse normal method was performed with metaRNAseq v. 1.0.8 package [99].

To analyze cell type composition of samples from different studies, we used the immunedeconv v. 2.1.0 package. Cell type proportions were inferred from combined TPM-normalized data from all samples using the quanTIseq method [100].

For visualization on PCA biplots, counts were transformed using variance stabilizing transformation as implemented in DESeq2 v. 1.44.0 [101] and batch-corrected with ComBat-seq [102] as implemented in the sva package v. 3.52.0.

For every type of analysis, *p*-values were corrected for multiple comparisons using the Benjamini–Hochberg (BH) procedure.

### 4.8. Data Availability

Bulk RNA sequencing raw data, counts, and TPMs generated in this study are deposited in Gene Expression Omnibus under GSE280402.

Code for data analysis is available at https://github.com/castrofiber/T2D_blood_meta, accessed on 18 November 2025.

## 5. Conclusions

Overall, our results corroborate earlier studies of type 2 diabetes pathology and pinpoint crucial genes and pathways that might serve as reproducible and reliable biomarkers of diabetes. However, low concordance between individual studies included in our analysis, as well as a substantial influence of various factors (such as blood cell composition) on the gene expression pattern of whole blood, indicates that any models proposed based on a single study of blood transcriptomes should be approached with caution, especially when translating research results into clinical practice.

## Figures and Tables

**Table 1:** RNA-seq datasets used in this paper.

Accession	Citation	Samples Healthy	Samples T2D	Samples Healthy After Filtering Outliers	Samples T2D After Filtering Outliers
GSE280402	this study	8	8	7	7
GSE154881	no	5	5	5	5
GSE153315	no	10	20	9	14
GSE184050	[4]	66	50	53	35
GSE221521	[5]	50	74	45	61
GSE185011	[6]	5	5	4	3
GSE181143	[7]	294	259	259	234
GSE114192	[8]	82	113	75	99
Total		520	534	457	458

**Table 2:** Designs for RNA-seq datasets used in this paper and number of DEGs for the main contrast.

Accession	Formula	# of DEGs
GSE280402	~0 + condition + sex	0
GSE154881	~0 + condition + sex	3417
GSE153315	~0 + condition + sex	0
GSE184050	~0 + condition + sex + timepoint + (1|individual)	1
GSE221521	~0 + condition + sex	0
GSE185011	~0 + condition + sex	0
GSE181143	~0 + condition + sex + tuberculosis_status + timepoint + site + (1|individual)	142
GSE114192	~0 + condition + sex + tuberculosis_status + site	3023

**Table 3:** Clinical characteristics of T2D patients and controls.

Characteristics	T2D (N = 8)	Controls (N = 8)	*p*-Value
Age (years)	66.3	56.6	0.35
Female (n)	5	5	NA
Male (n)	4	4	NA
Family history (n)	1	5	NA
Fasting blood glucose *	8.8	4.7	0.0096
Total cholesterol (mmol/L)	5.8	6.7	0.33
HDL (mmol/L)	1.5	1.6	0.75
LDL (mmol/L)	3	4.4	0.25
Creatinine (mmol/L)	0.1	0.08	0.14
BMI (kg/m^2^) *	32	24.5	0.0007
WHR *	1	0.76	0.01

* *p* < 0.05 compared to the control group; NA—not applicable; Family history—family history of T2D in close relatives.

## Data Availability

Bulk RNA sequencing raw data, counts, and TPMs generated in this study are deposited under GSE280402.

## Supplementary Materials

The following supporting information can be downloaded at https://www.mdpi.com/article/10.3390/ijms262412046/s1.

## Abbreviations

The following abbreviations are used in this manuscript

T2D	Type 2 diabetes
DEG	Differentially expressed gene
IDD	Integration-driven discovery
PBMC	Peripheral blood mononuclear cells
GO	Gene Ontology
GEO	Gene Expression Omnibus
ERAD	Endoplasmic-reticulum-associated protein degradation
FBG	Fasting blood glucose
PCA	Principal component analysis
HCA	Hierarchical cluster analysis
CCC	Cophenetic correlation coefficient
