# Supplementary material for: Cross-Study Meta-Analysis of Blood Transcriptomes in Type 2 Diabetes
Source: Int J Mol Sci. 2025 Dec 15;26(24):12046. doi: 10.3390/ijms262412046 (PMC12732418; doi:10.3390/ijms262412046)
Supplement: Supplementary file 1 [file ijms-26-12046-s001.zip › S14.pdf]

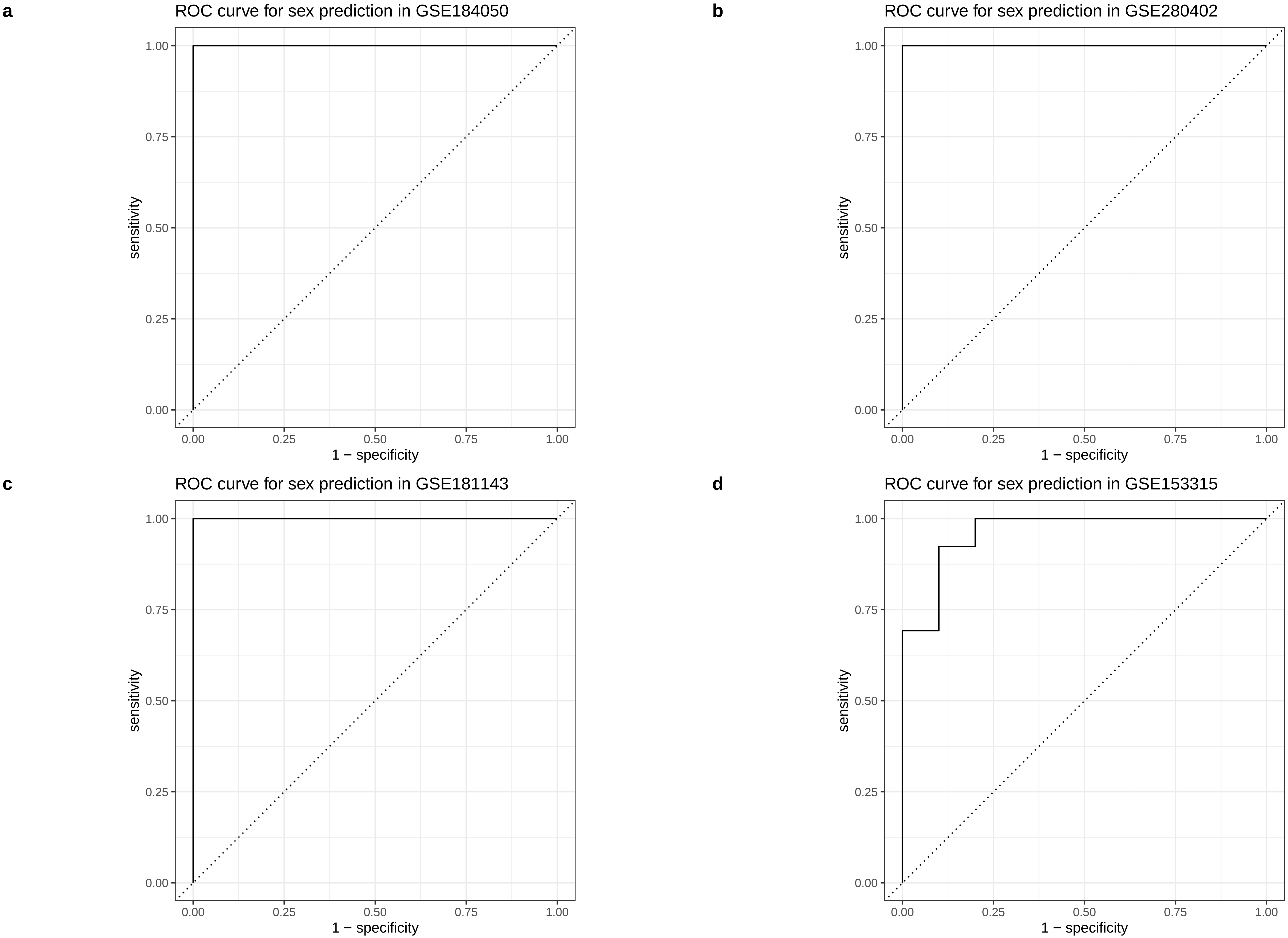
a
ROC curve for sex prediction in GSE184050
b
ROC curve for sex prediction in GSE280402
c
ROC curve for sex prediction in GSE181143
d
ROC curve for sex prediction in GSE153315
sensitivity
1 − specificity
0.00
0.25
0.50
0.75
1.00
