# Supplementary material for: Cross-Study Meta-Analysis of Blood Transcriptomes in Type 2 Diabetes
Source: Int J Mol Sci. 2025 Dec 15;26(24):12046. doi: 10.3390/ijms262412046 (PMC12732418; doi:10.3390/ijms262412046)

Variance−stabilized expression of *FBLN2*, *TPCN1*, *PC*, *SHANK1*, and *PLD4* in all samples

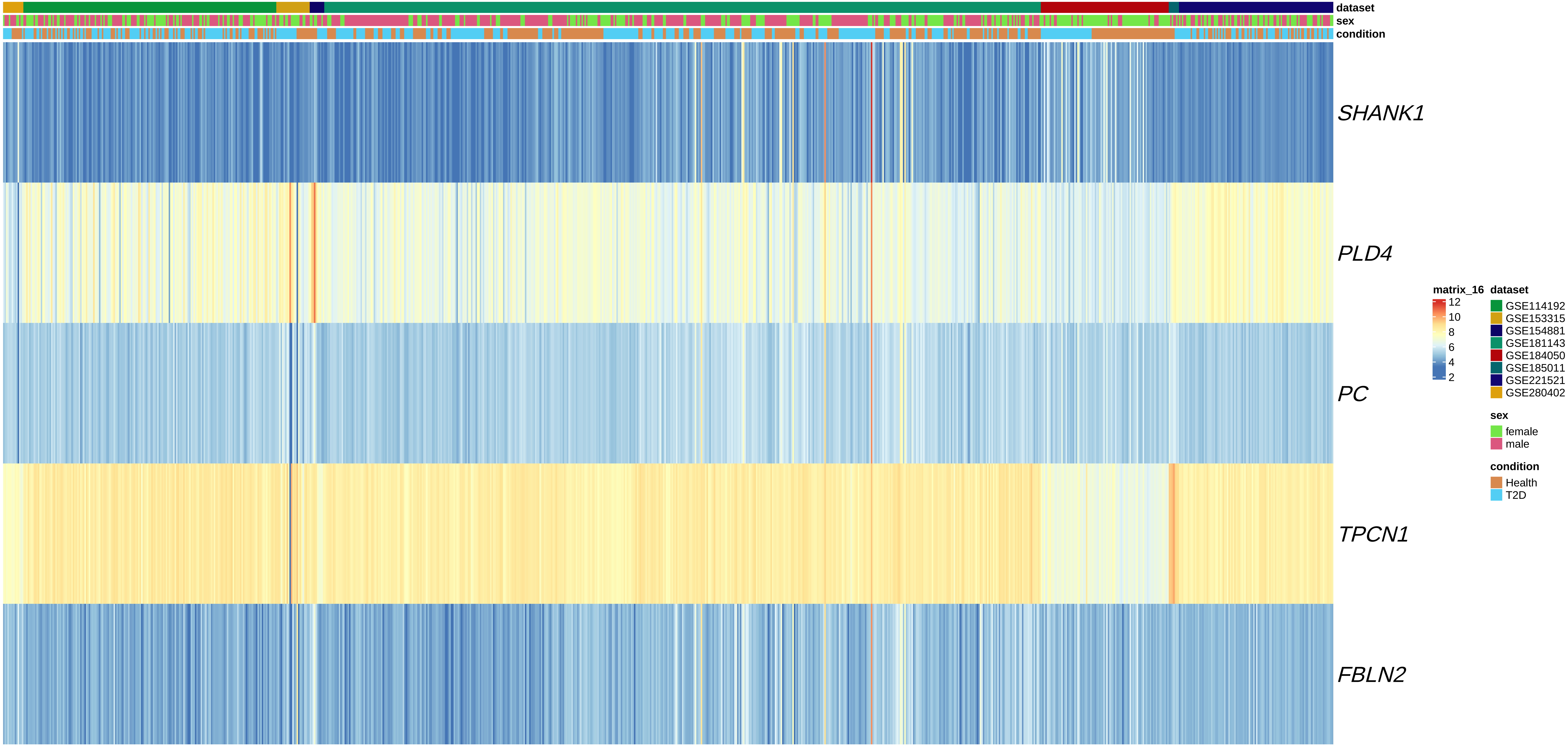

Supplement: Supplementary file 1 [file ijms-26-12046-s001.zip › S9.pdf]
