# Supplementary material for: Cross-Study Meta-Analysis of Blood Transcriptomes in Type 2 Diabetes
Source: Int J Mol Sci. 2025 Dec 15;26(24):12046. doi: 10.3390/ijms262412046 (PMC12732418; doi:10.3390/ijms262412046)
Supplement: Supplementary file 1 [file ijms-26-12046-s001.zip › Supplementary figures.pdf]

Supplementary figures

S1-8. Differential expression analysis results for individual datasets. A. MA plot, B. PCA biplot colored based on diabetes status C. Variance plot for all parameters in the formula for analysis and residuals.

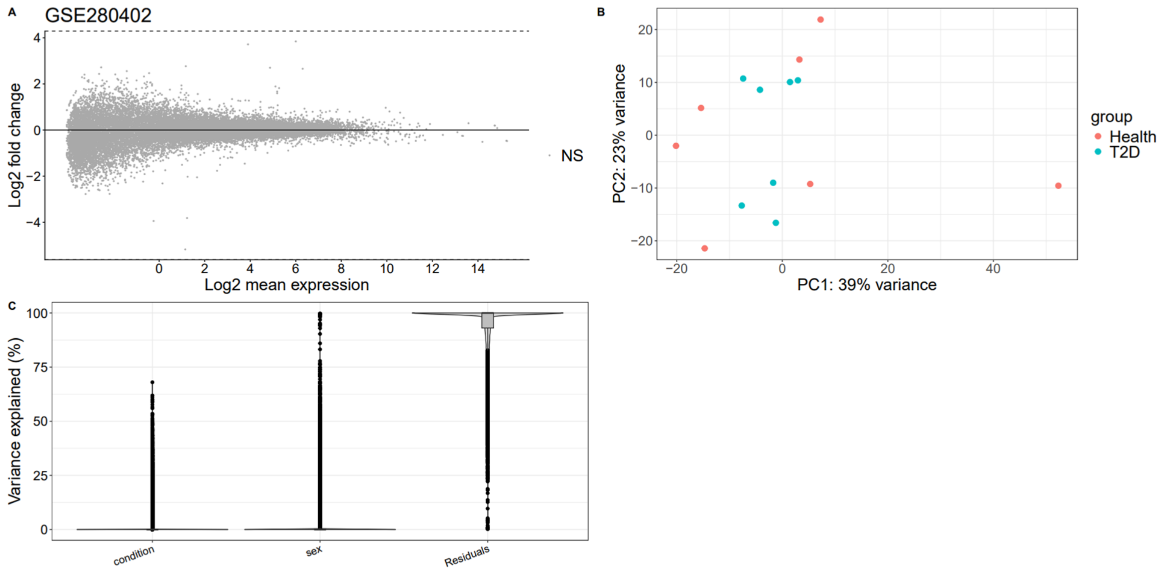

Supplementary figure 1. Differential expression analysis results GSE280402. A. MA plot, B. PCA biplot colored based on diabetes status C. Variance plot for all parameters in the formula for analysis and residuals.

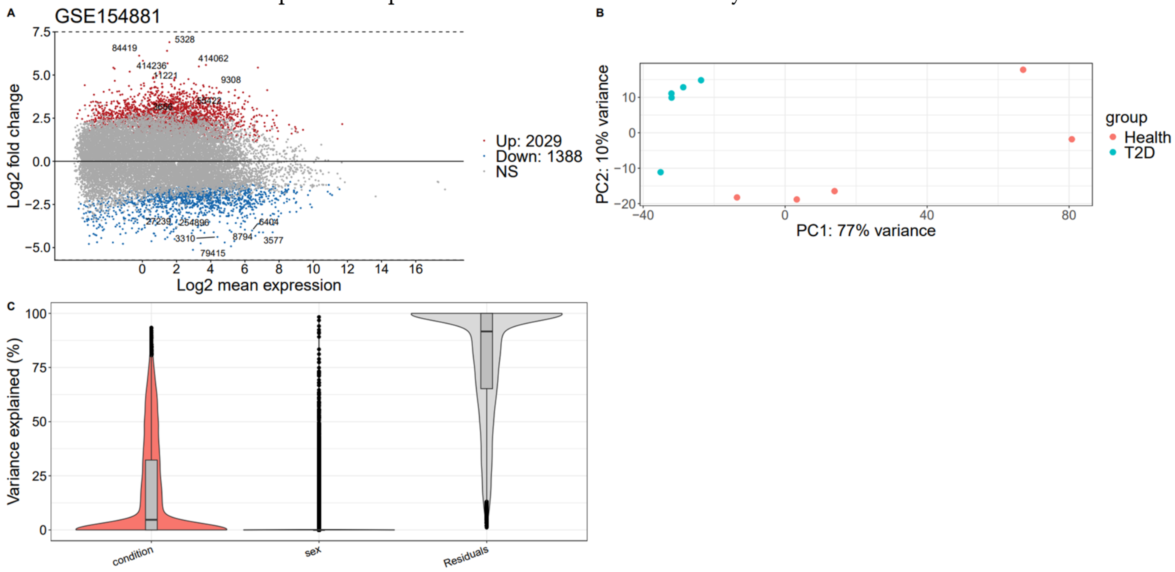

Supplementary figure 2. Differential expression analysis results GSE154881. A. MA plot, B. PCA biplot colored based on diabetes status C. Variance plot for all parameters in the formula for analysis and residuals.

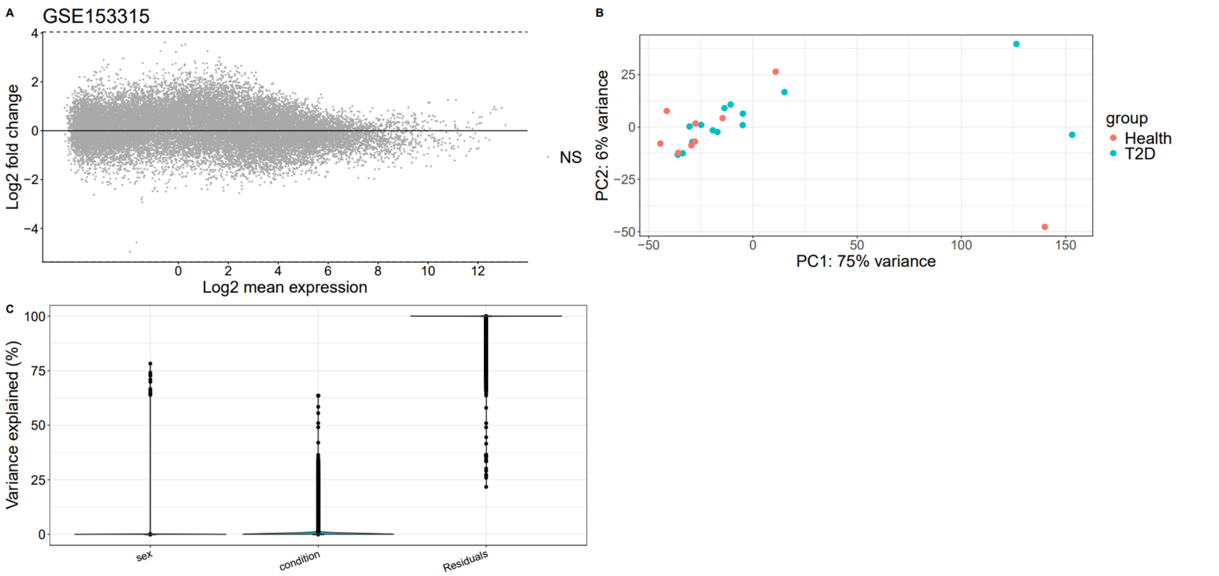

Supplementary figure 3. Differential expression analysis results GSE153315. A. MA plot, B. PCA biplot colored based on diabetes status C. Variance plot for all parameters in the formula for analysis and residuals.

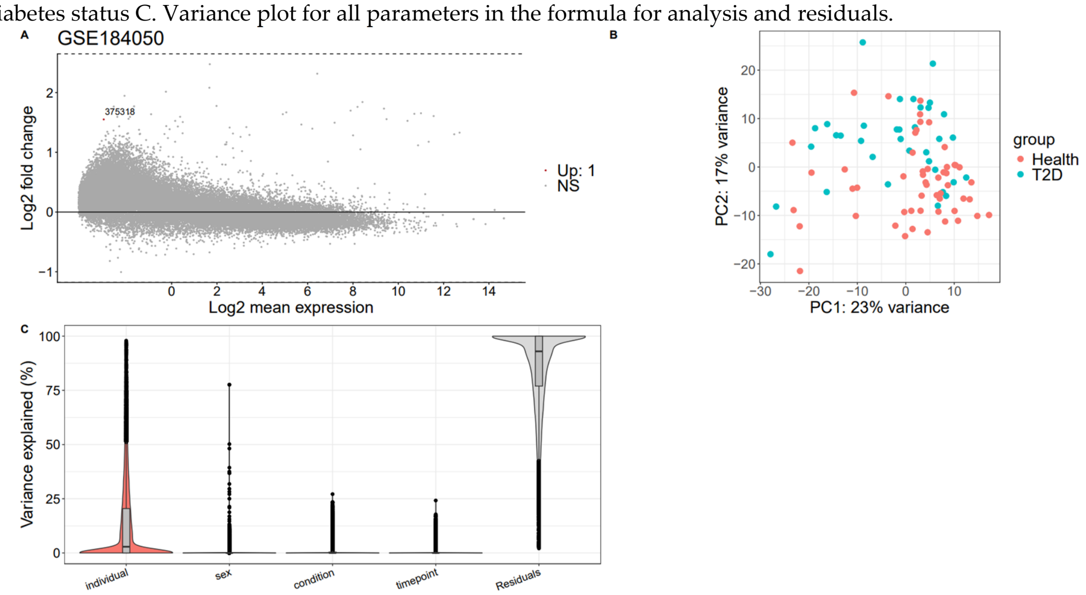

Supplementary figure 4. Differential expression analysis results GSE184050. A. MA plot, B. PCA biplot colored based on diabetes status C. Variance plot for all parameters in the formula for analysis and residuals.

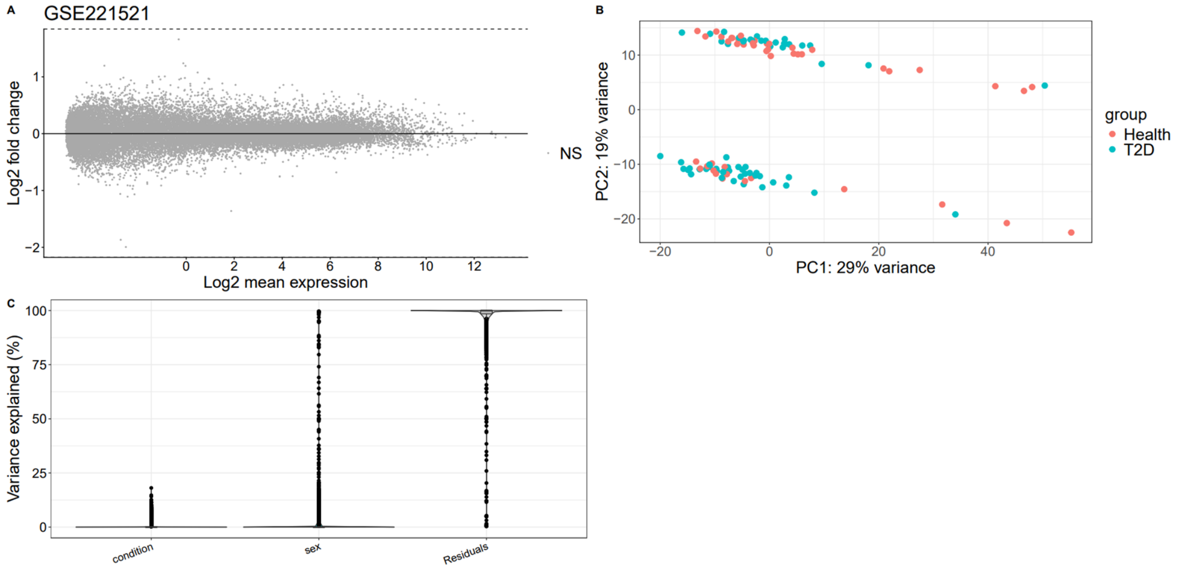

Supplementary figure 5. Differential expression analysis results GSE221521. A. MA plot, B. PCA biplot colored based on diabetes status C. Variance plot for all parameters in the formula for analysis and residuals.

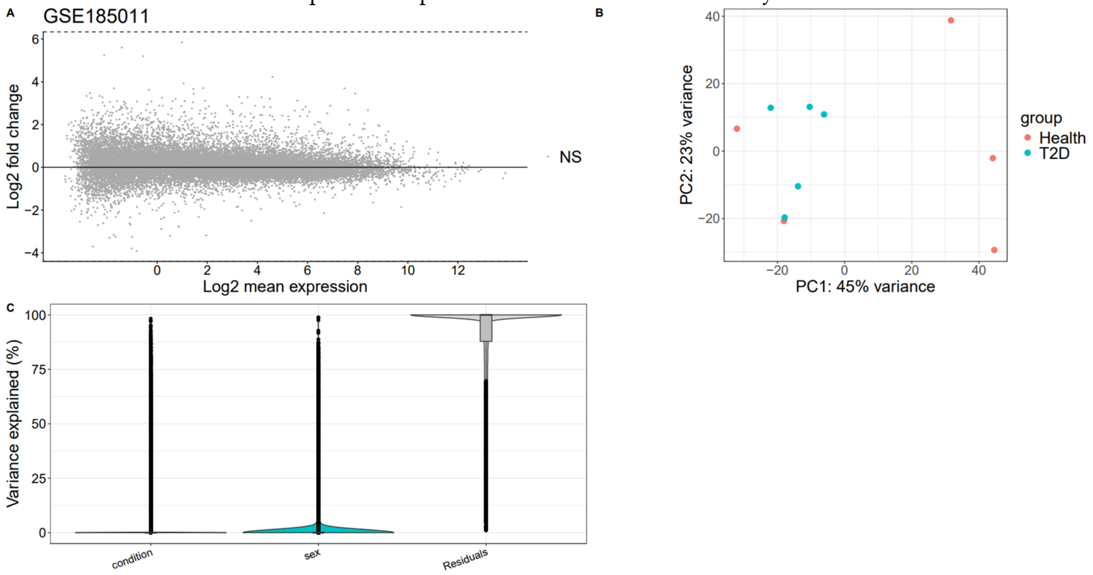

Supplementary figure 6. Differential expression analysis results GSE185011. A. MA plot, B. PCA biplot colored based on diabetes status C. Variance plot for all parameters in the formula for analysis and residuals.

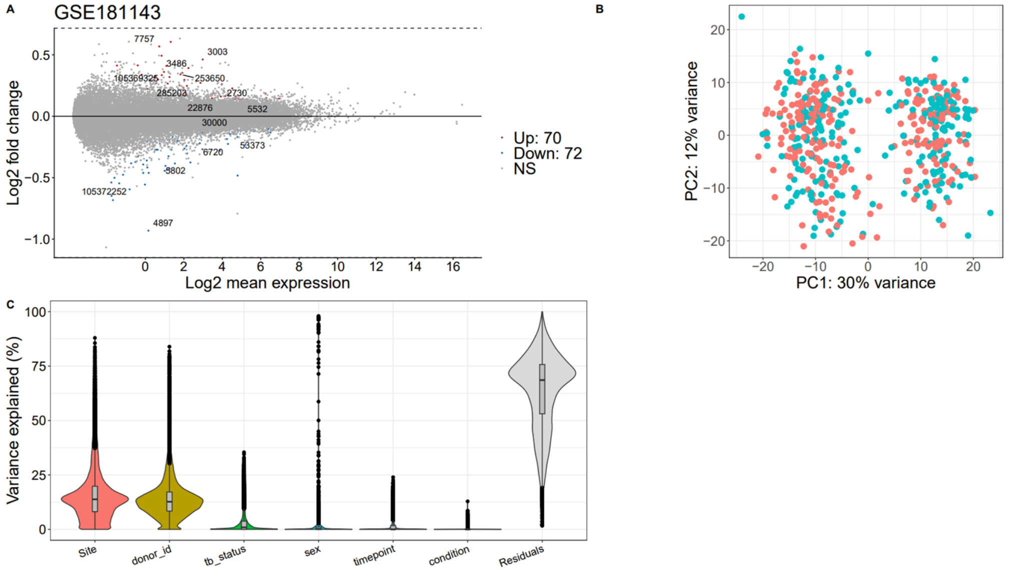

Supplementary figure 7. Differential expression analysis results GSE181143. A. MA plot, B. PCA biplot colored based on diabetes status C. Variance plot for all parameters in the formula for analysis and residuals.

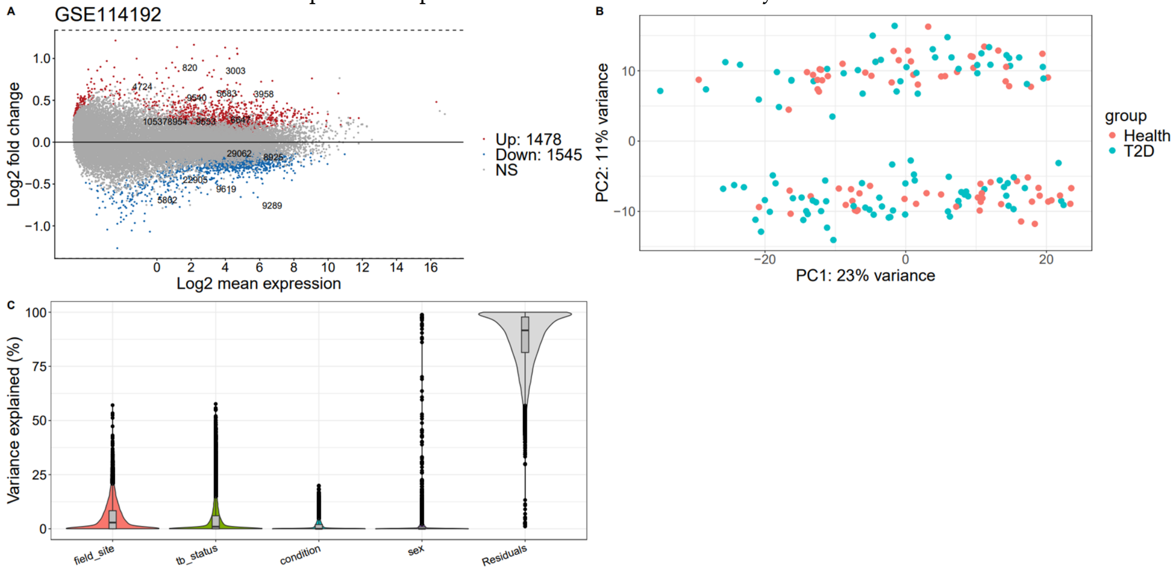

Supplementary figure 8. Differential expression analysis results GSE114192. A. MA plot, B. PCA biplot colored based on diabetes status C. Variance plot for all parameters in the formula for analysis and residuals.

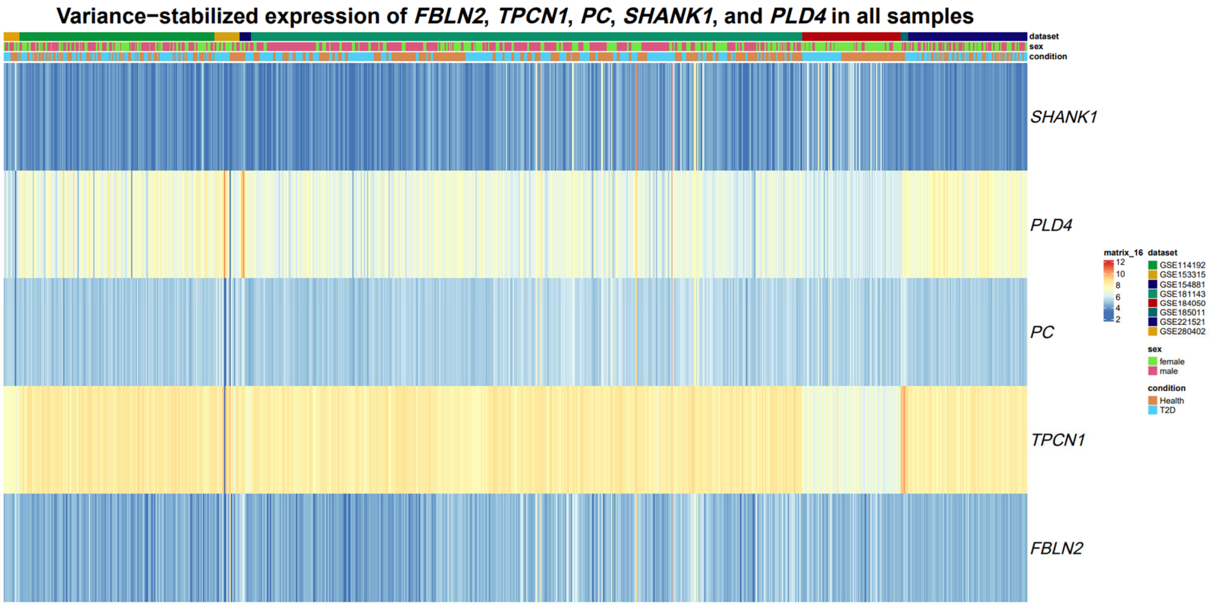

Supplementary figure 9. Variance-stabilized expression of *FBLN2*, *TPCN1*, *PC*, *SHANK1*, and *PLD4* in all samples. Colors on the top panel depict datasets, diabetes status and sex.

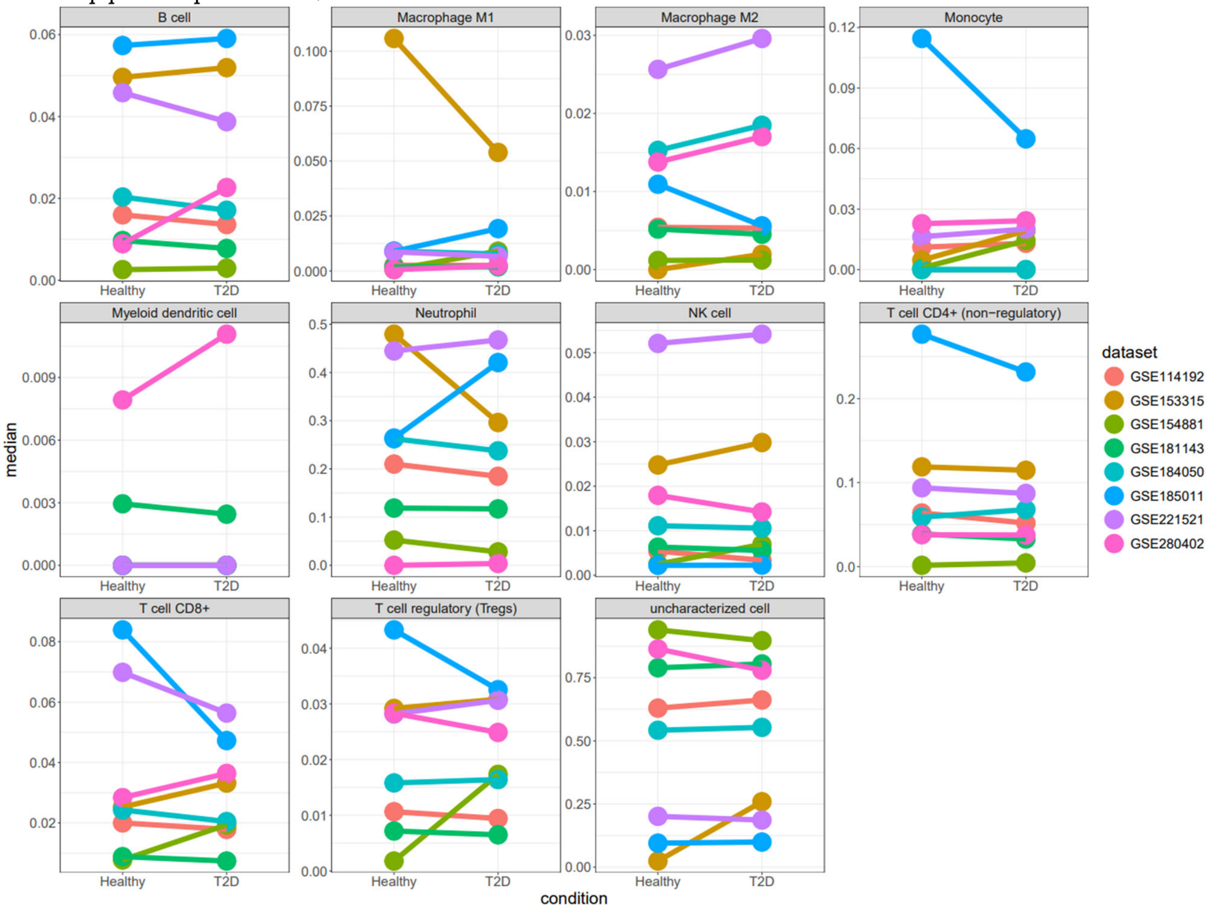

Supplementary figure 10. Dotplot of median cell-type proportions in different datasets for healthy and type 2 diabetes samples. T2D status is shown on the x axis, y axis demonstrates proportions, color and lines represent dots corresponding to the same dataset, and individual panels are showing proportions of different predicted cell types.

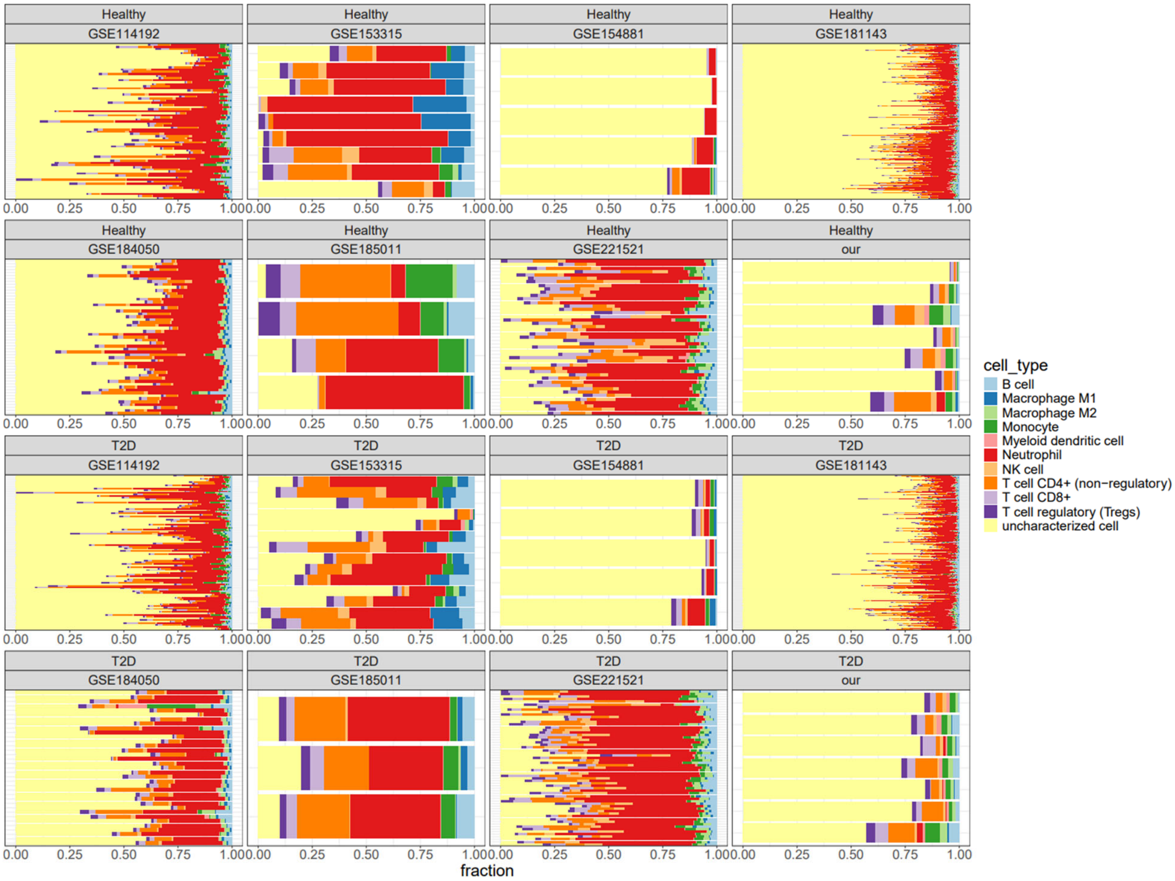

Supplementary figure 11. Deconvolution results by dataset and diabetes status

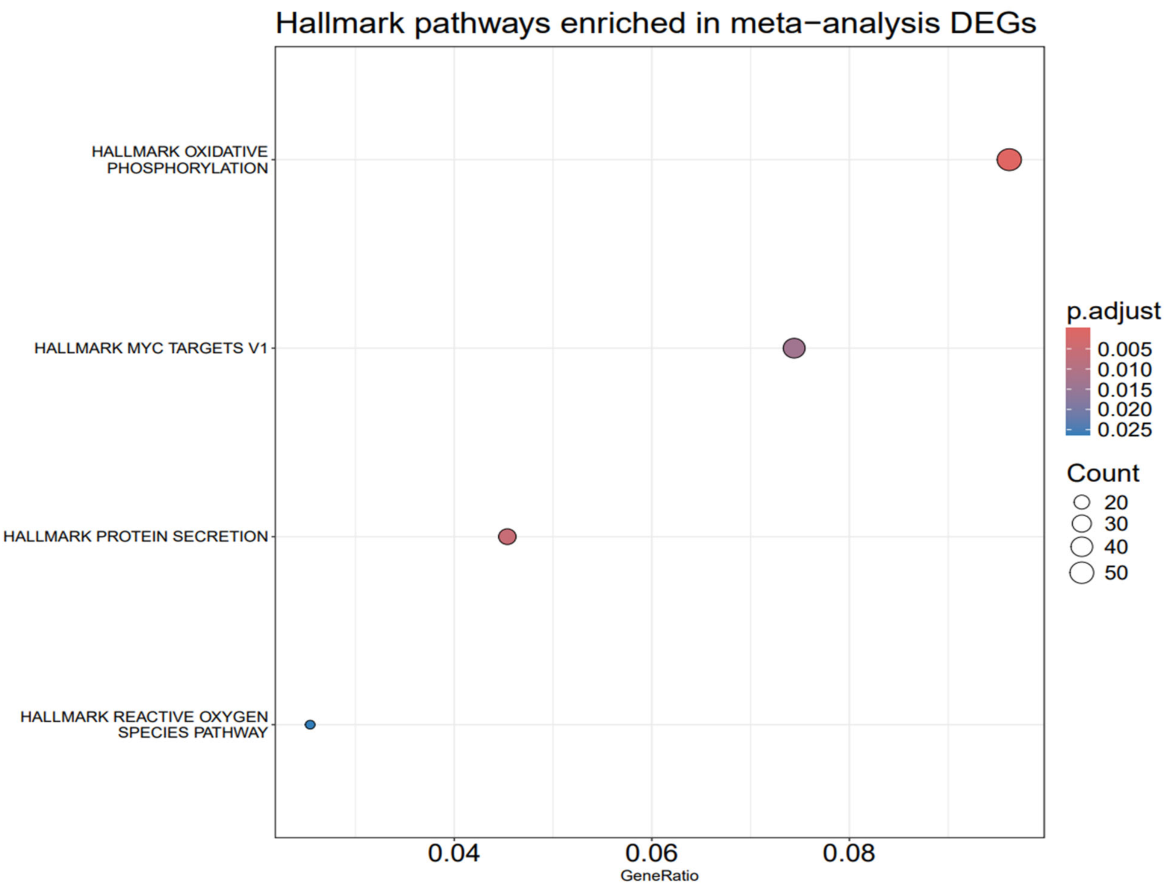

Supplementary figure 12. Enrichment of meta-analysis discoveries in Hallmark pathways.

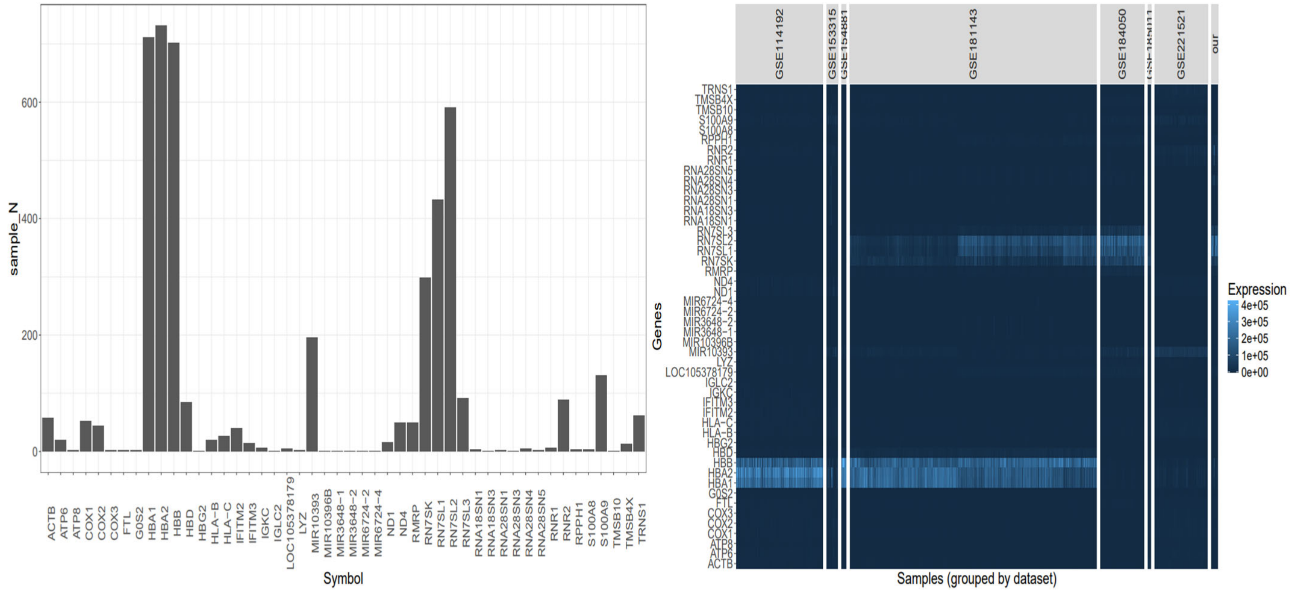

Supplementary figure 13. A. Top most popular most expressed genes (y axis; sample_N = how many samples have this gene in top5 expressed genes, x axis = gene symbol). B Heatmap for top5 gene expression (y axis – gene symbol, x axis = sample and dataset, color = expression in TPM)

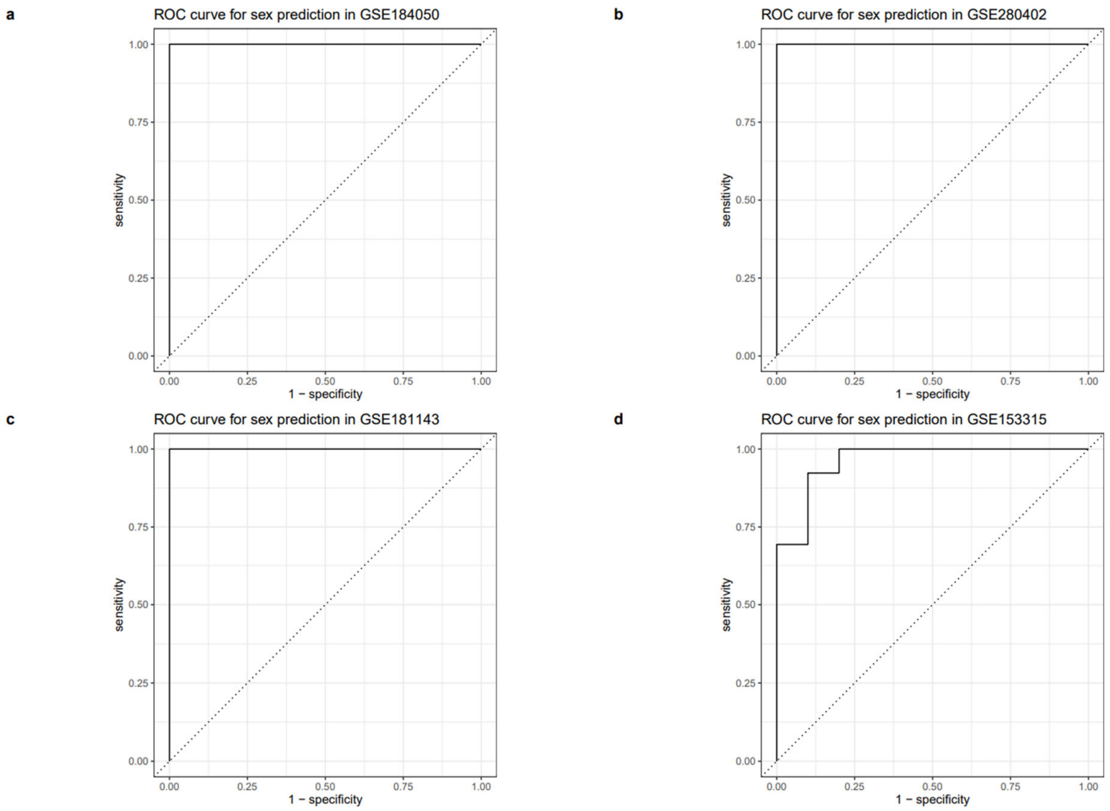

Supplementary figure 14. ROC curves for sex prediction in datasets with available sex metadata A. GSE184050 B. GSE280402 C. GSE181143 D. GSE153315.
