# Supplementary figures and images for: Cross-Study Meta-Analysis of Blood Transcriptomes in Type 2 Diabetes
Source: Int J Mol Sci. 2025 Dec 15;26(24):12046. doi: 10.3390/ijms262412046 (PMC12732418; doi:10.3390/ijms262412046)

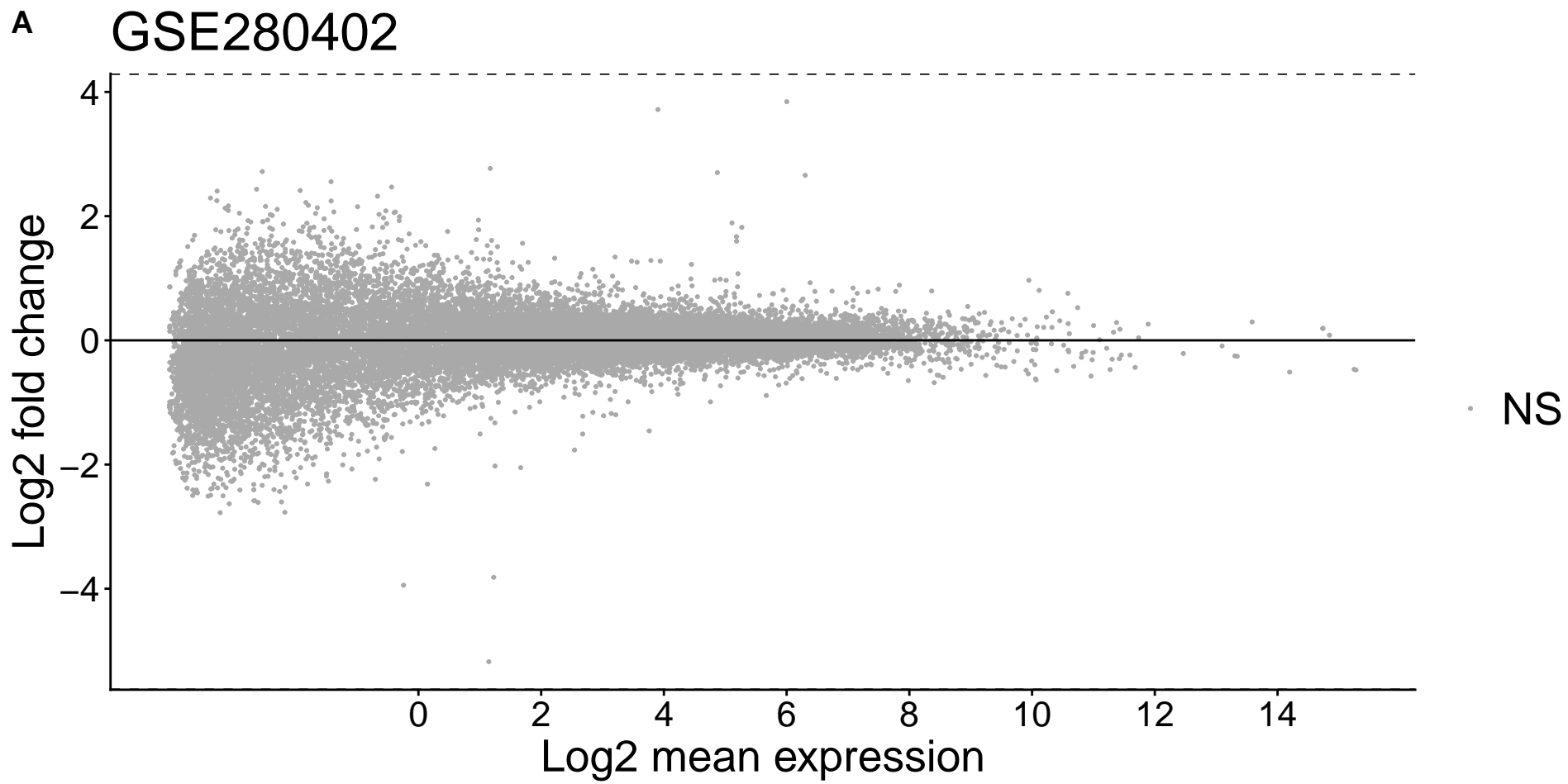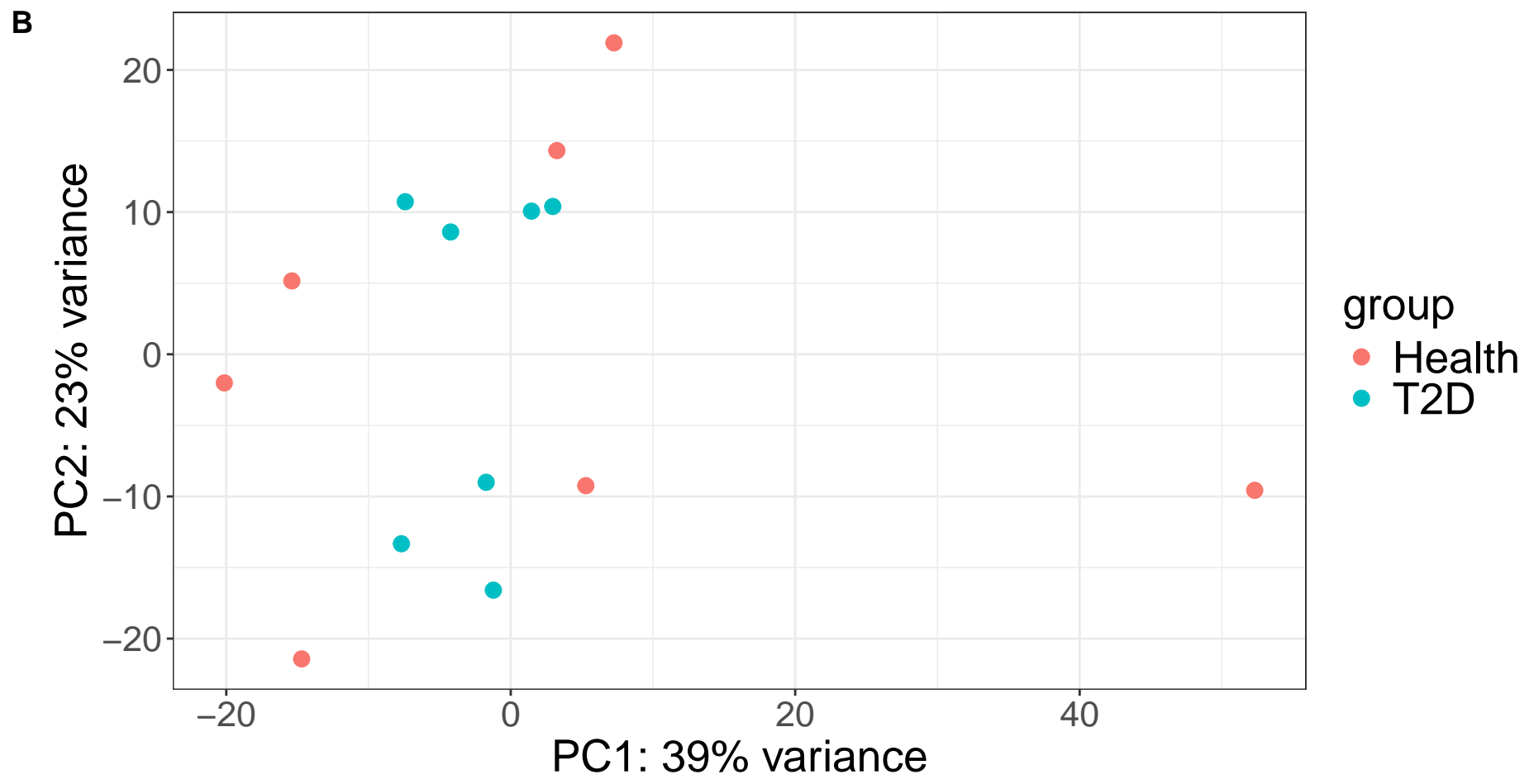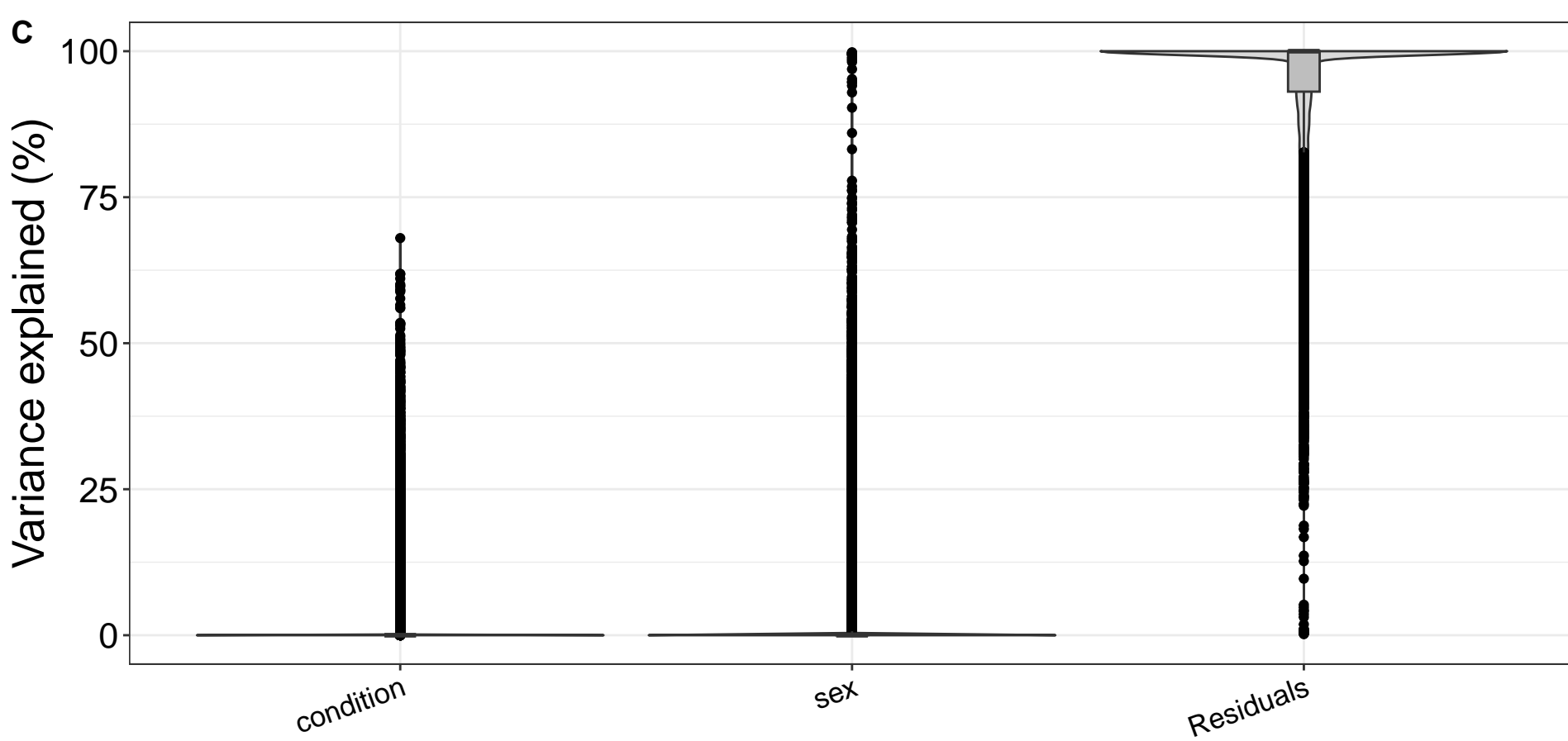

Supplement: Supplementary file 1 [file ijms-26-12046-s001.zip › S1.pdf]

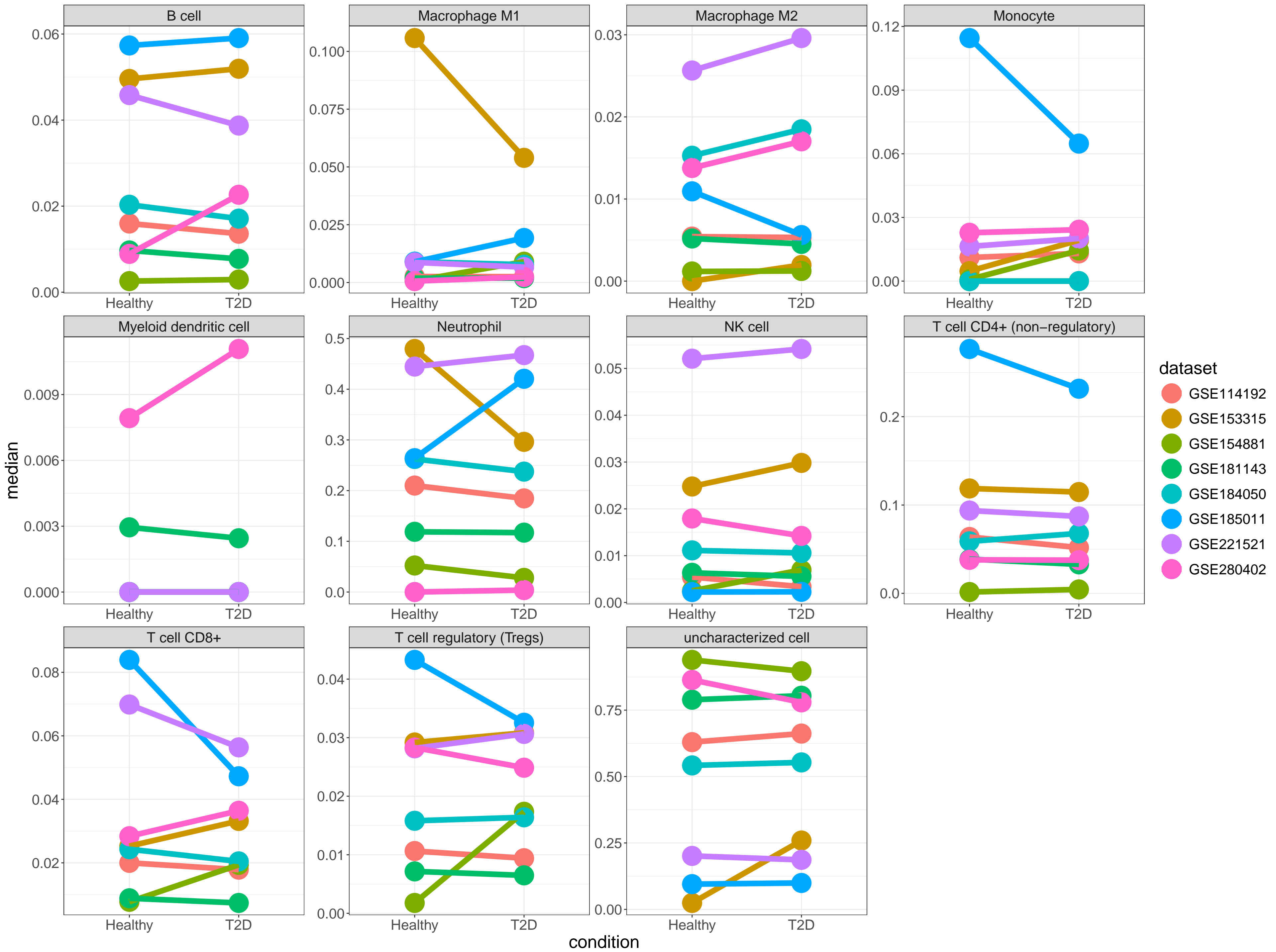

Supplement: Supplementary file 1 [file ijms-26-12046-s001.zip › S10.pdf]

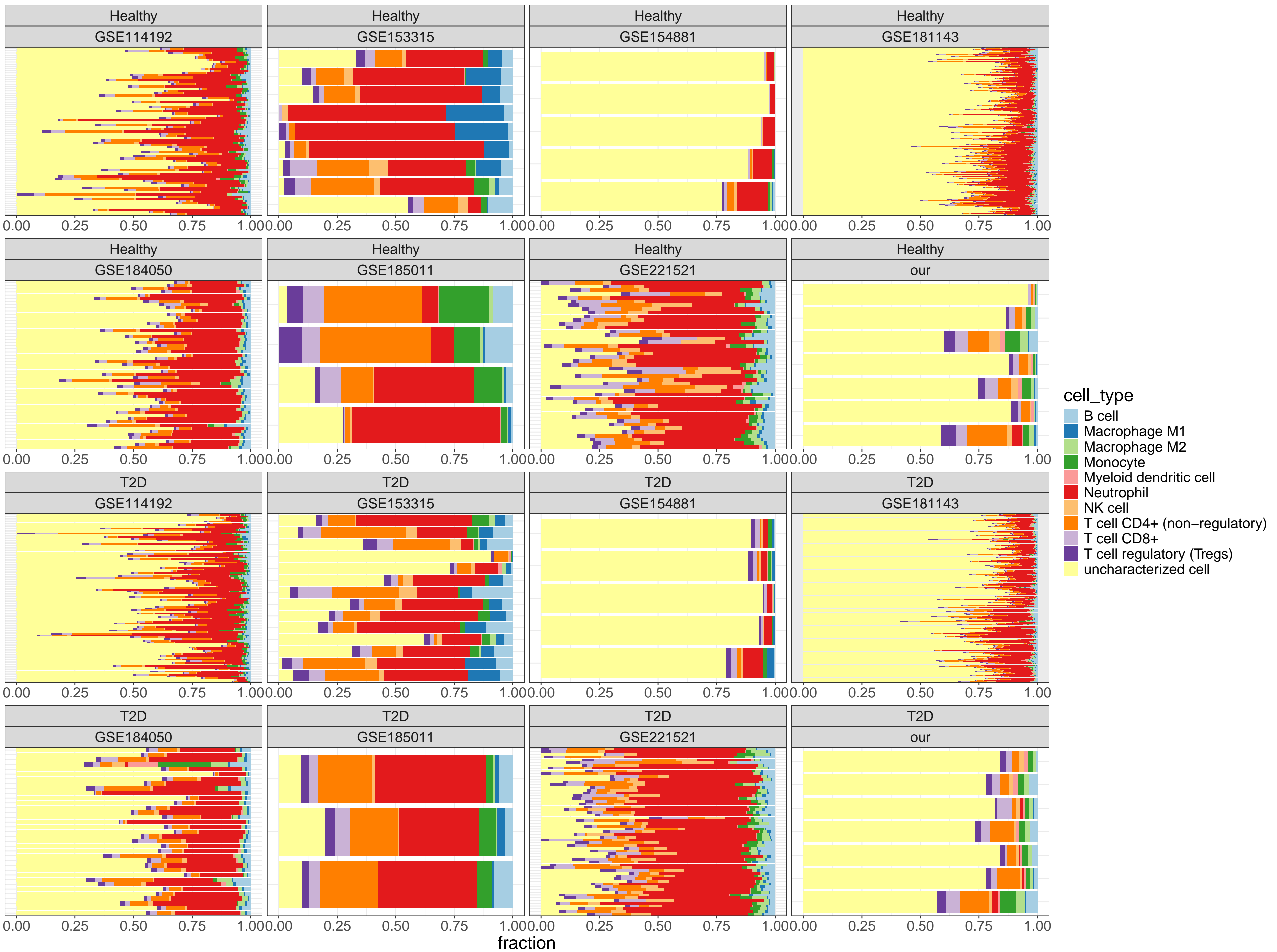

Supplement: Supplementary file 1 [file ijms-26-12046-s001.zip › S11.pdf]

# Hallmark pathways enriched in meta-analysis DEGs

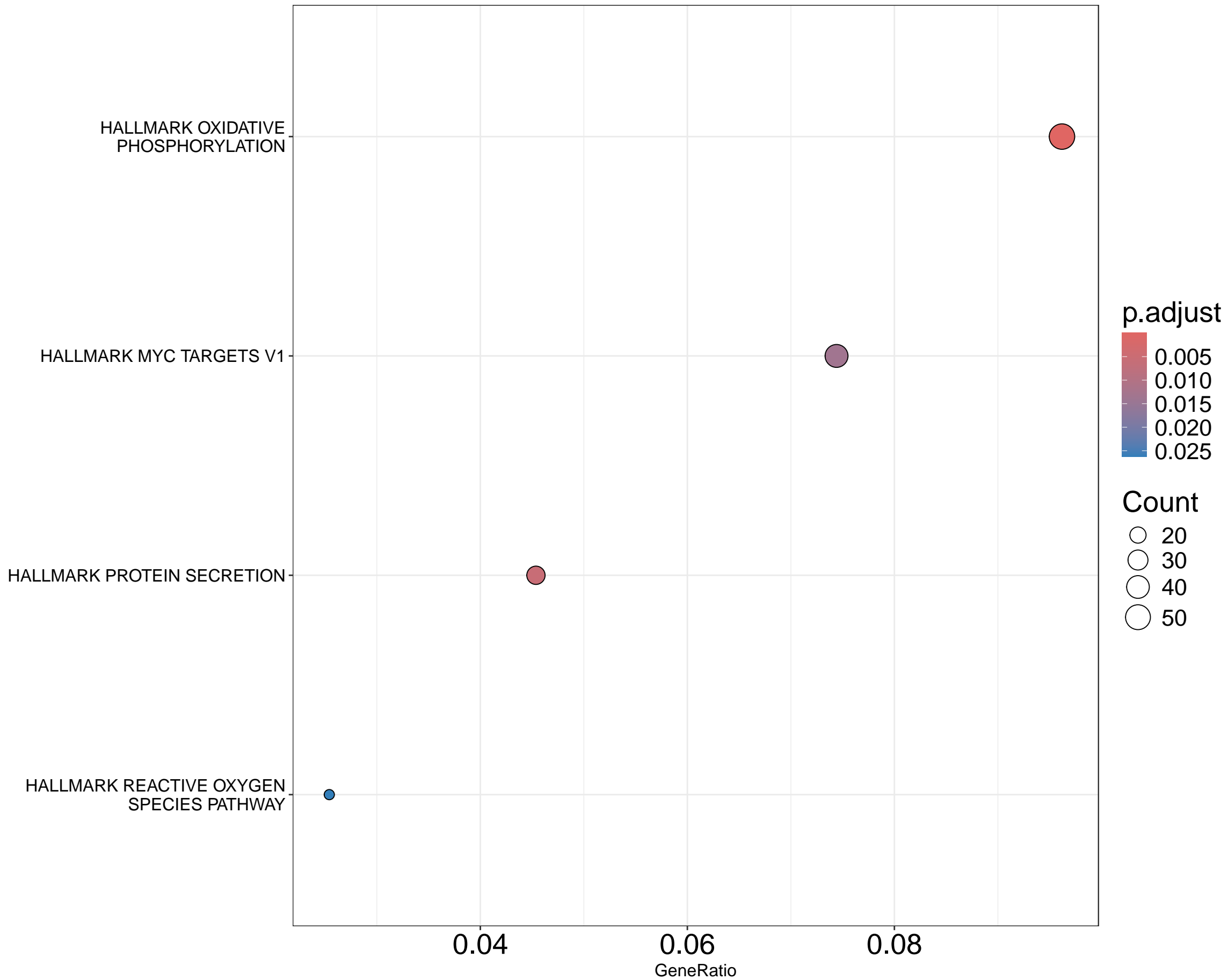

Supplement: Supplementary file 1 [file ijms-26-12046-s001.zip › S12.pdf]

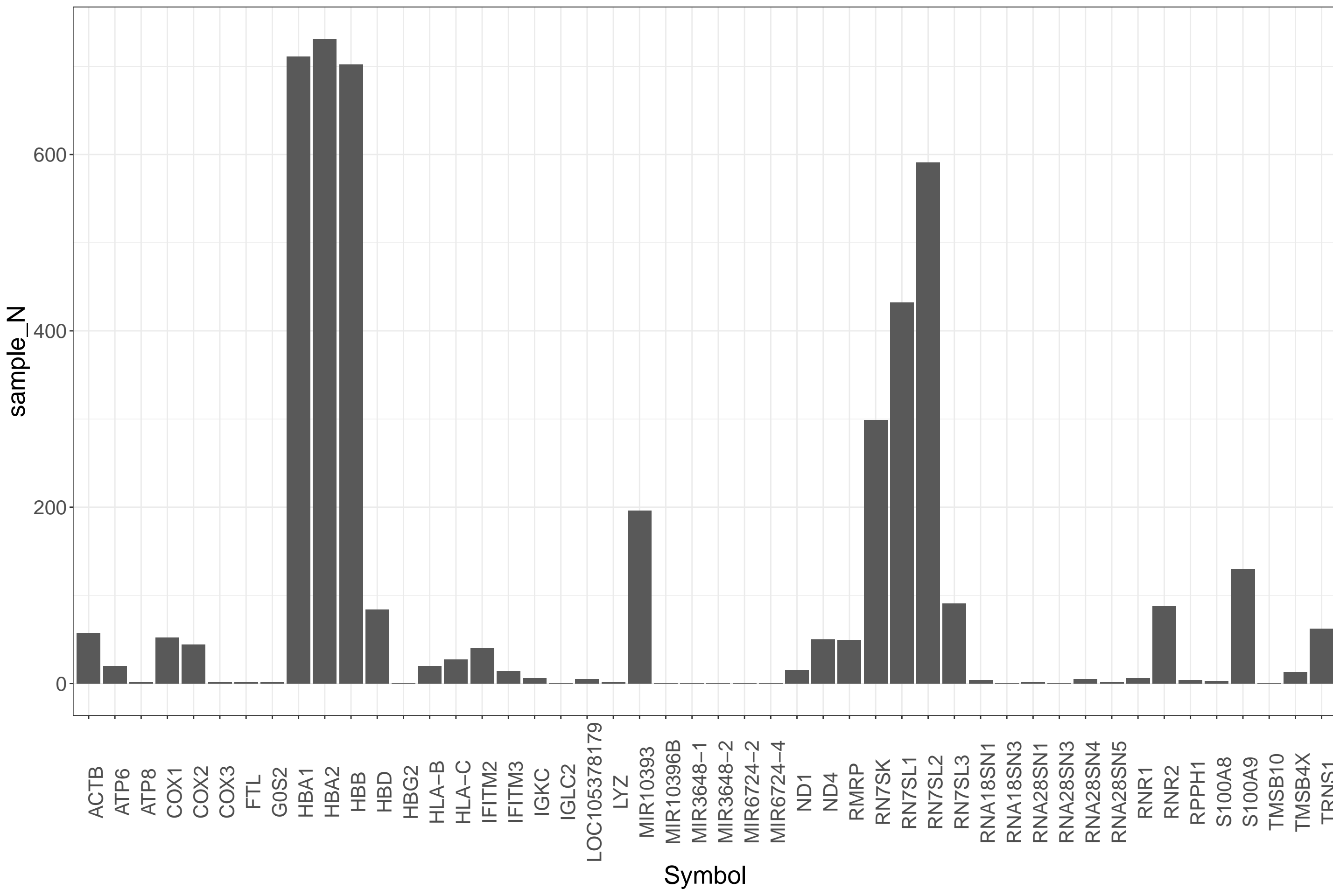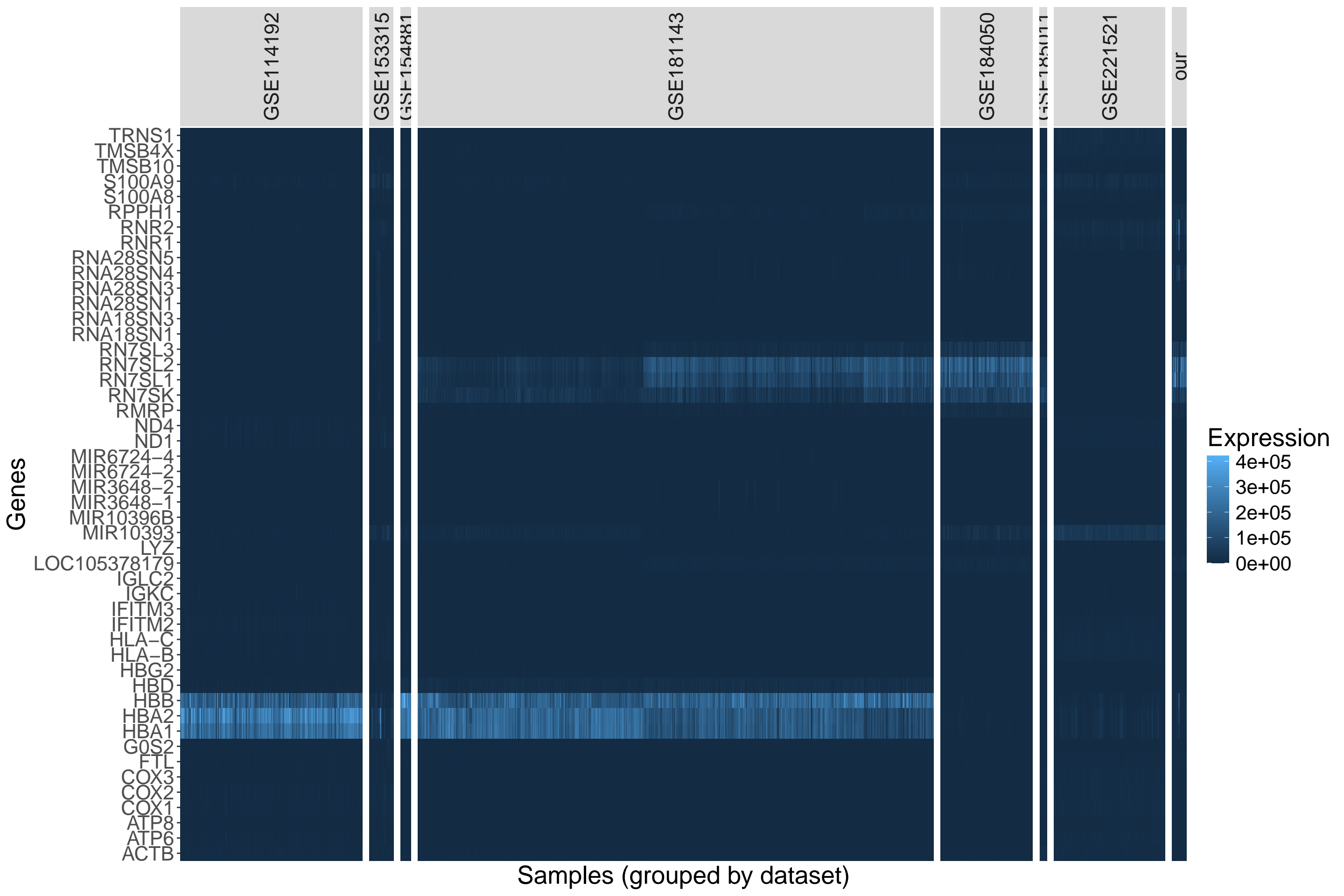

Supplement: Supplementary file 1 [file ijms-26-12046-s001.zip › S13.pdf]

A

GSE154881

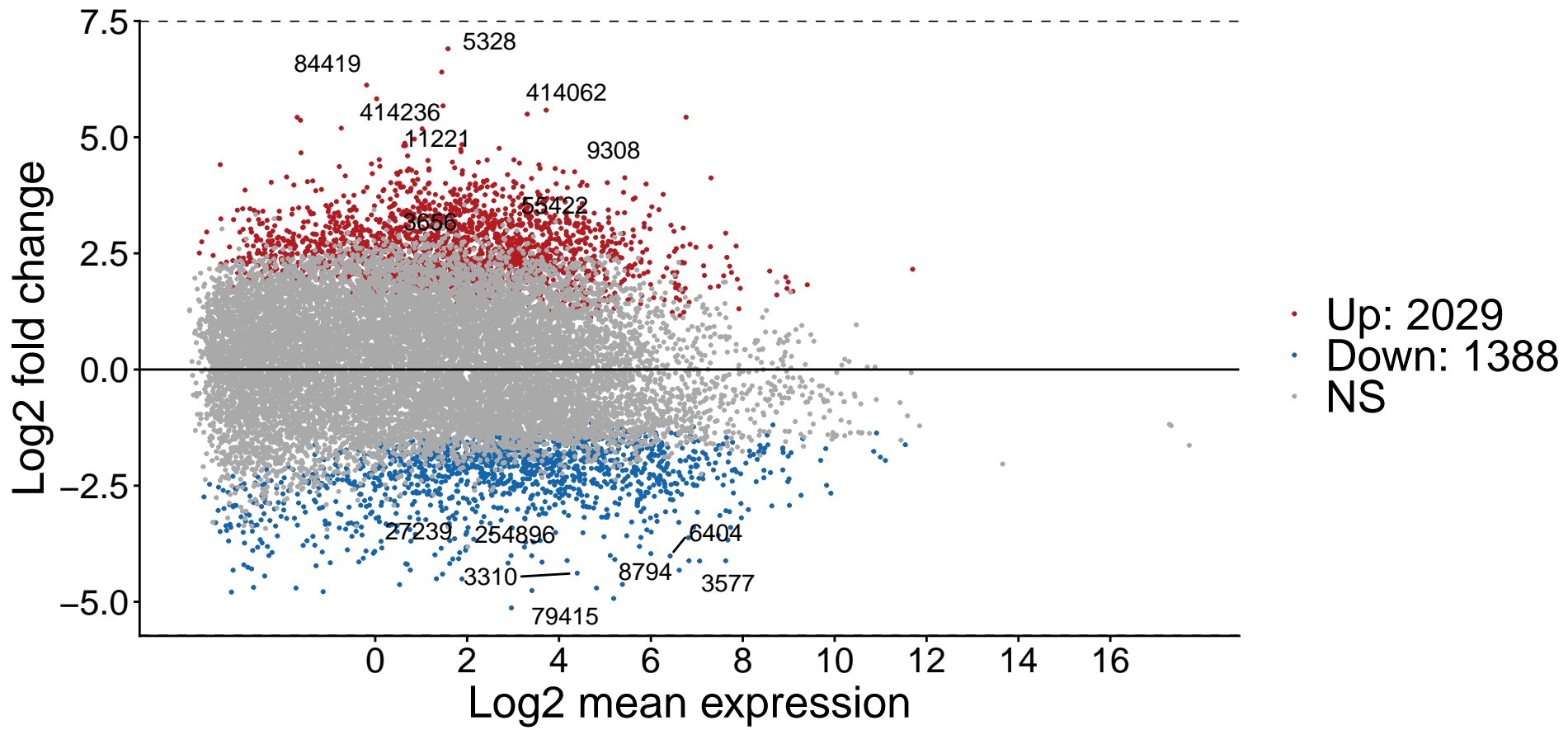

B

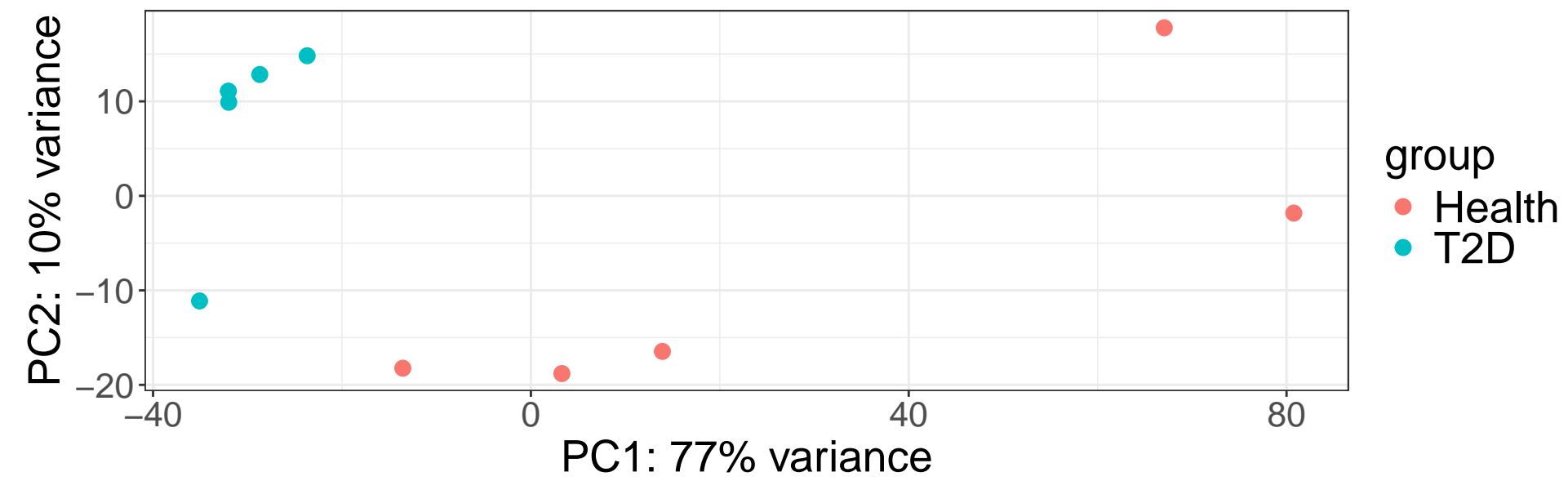

C

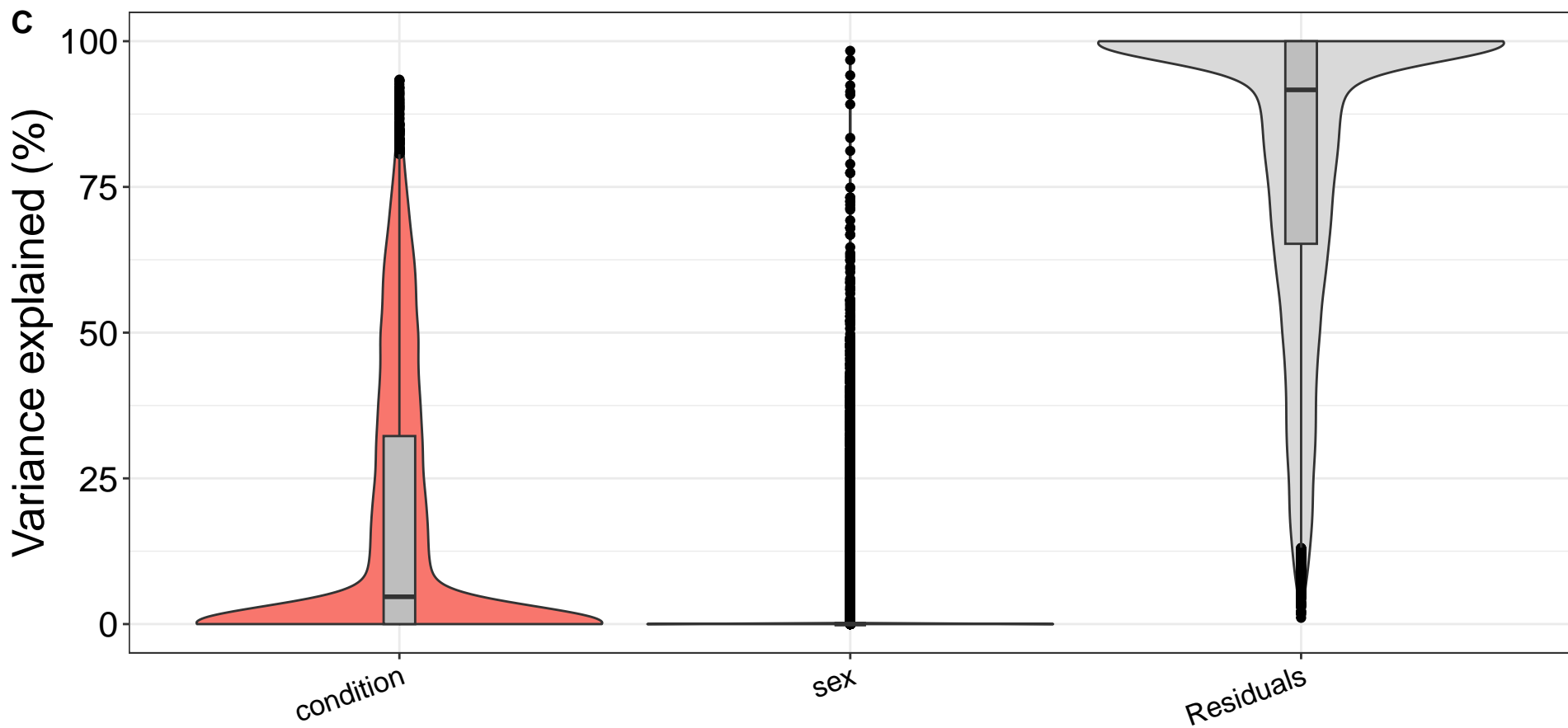

Supplement: Supplementary file 1 [file ijms-26-12046-s001.zip › S2.pdf]

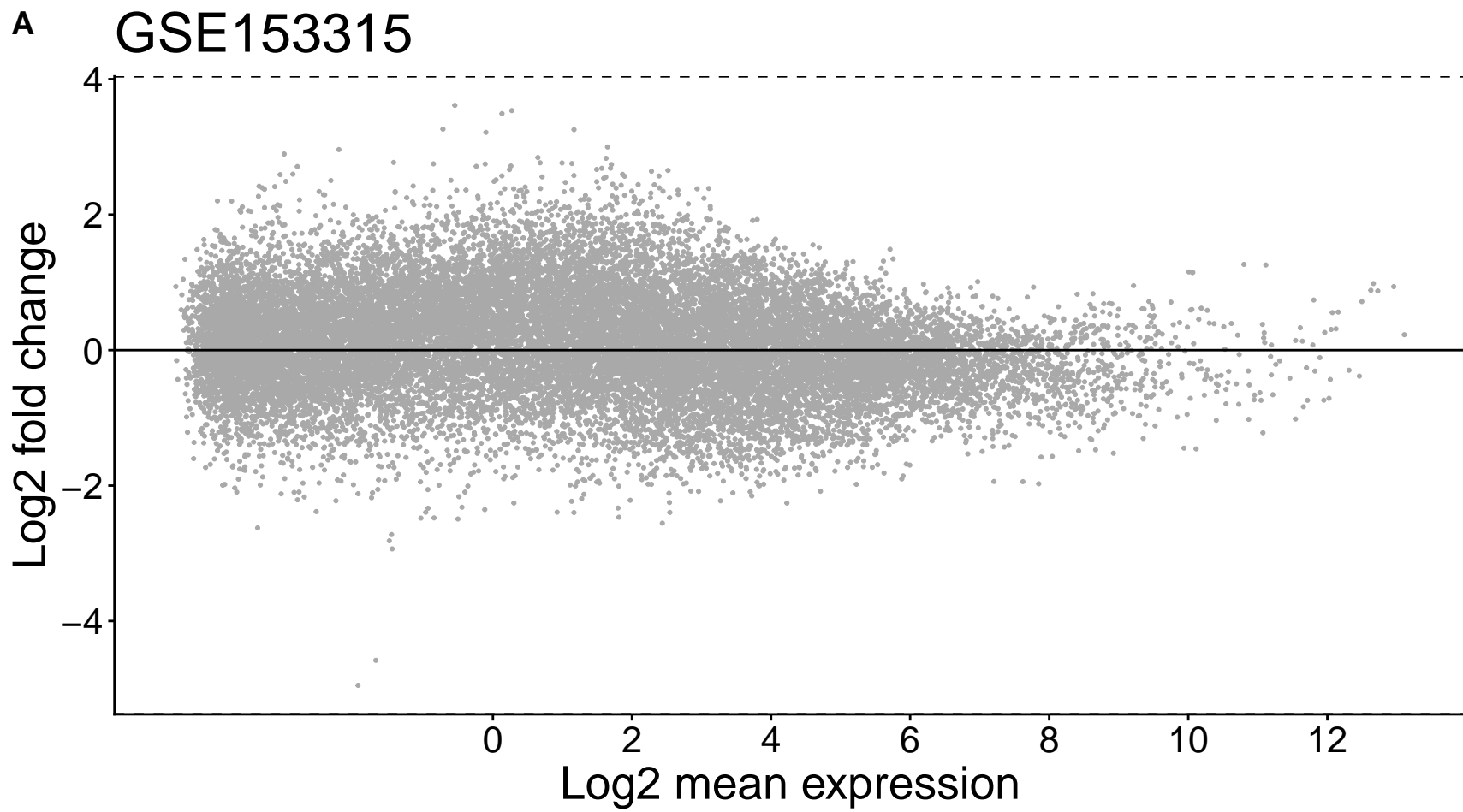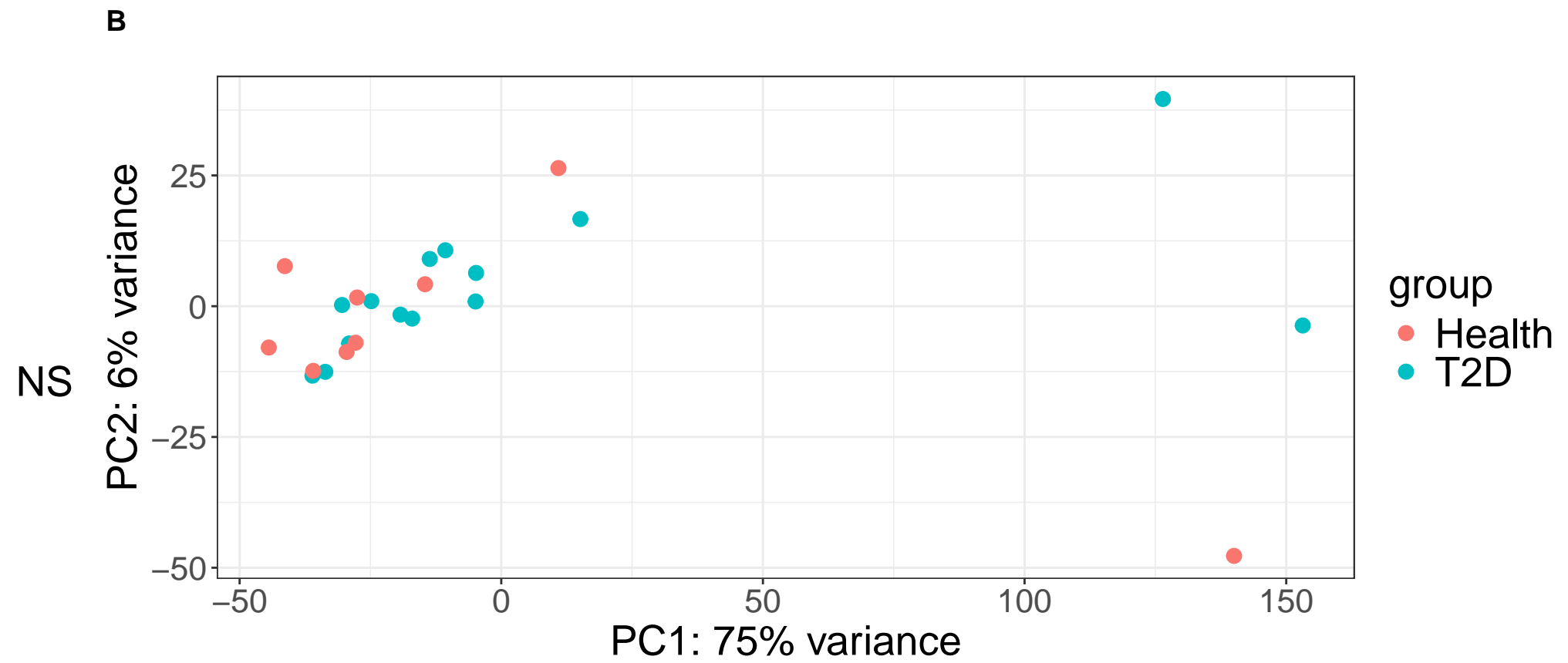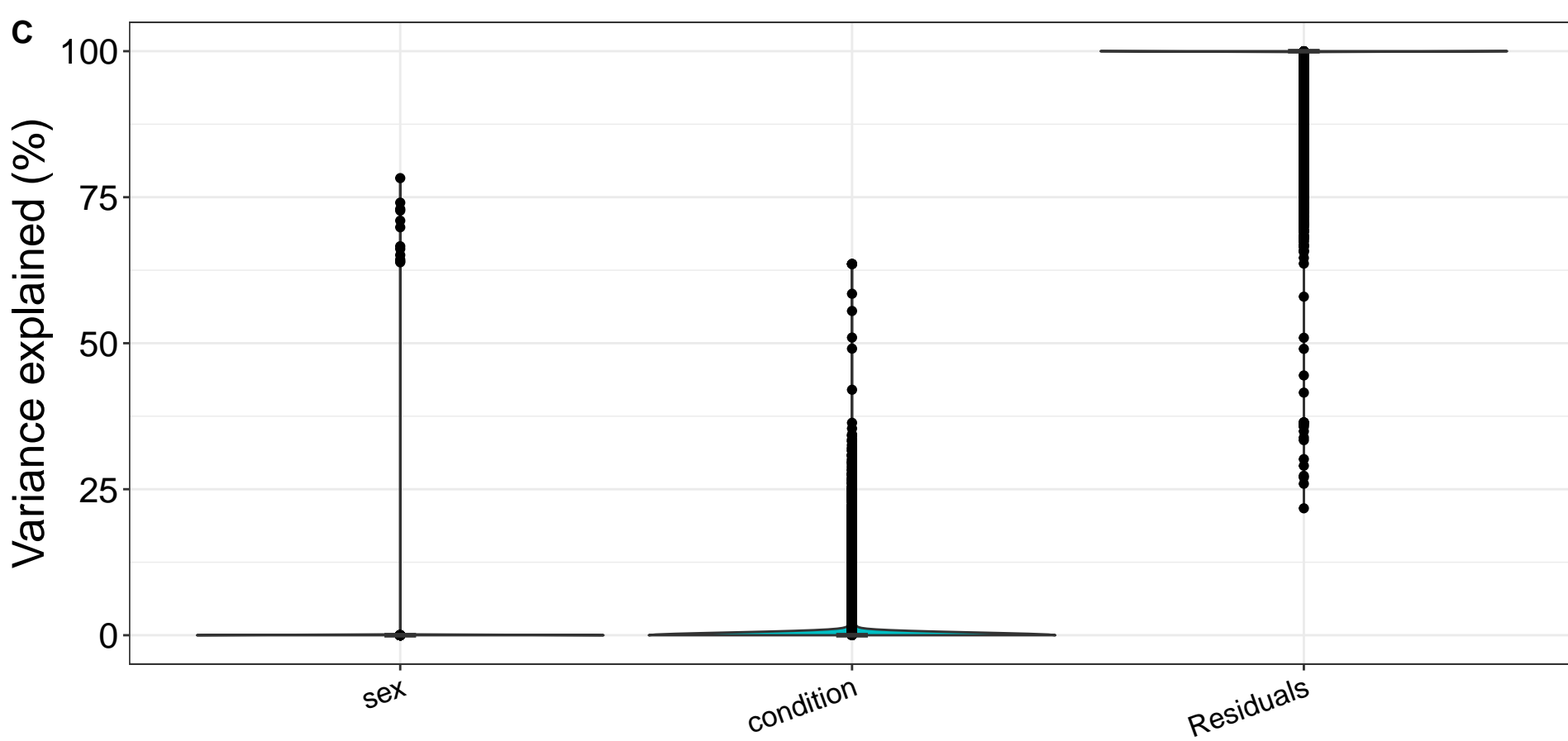

Supplement: Supplementary file 1 [file ijms-26-12046-s001.zip › S3.pdf]

**A** GSE184050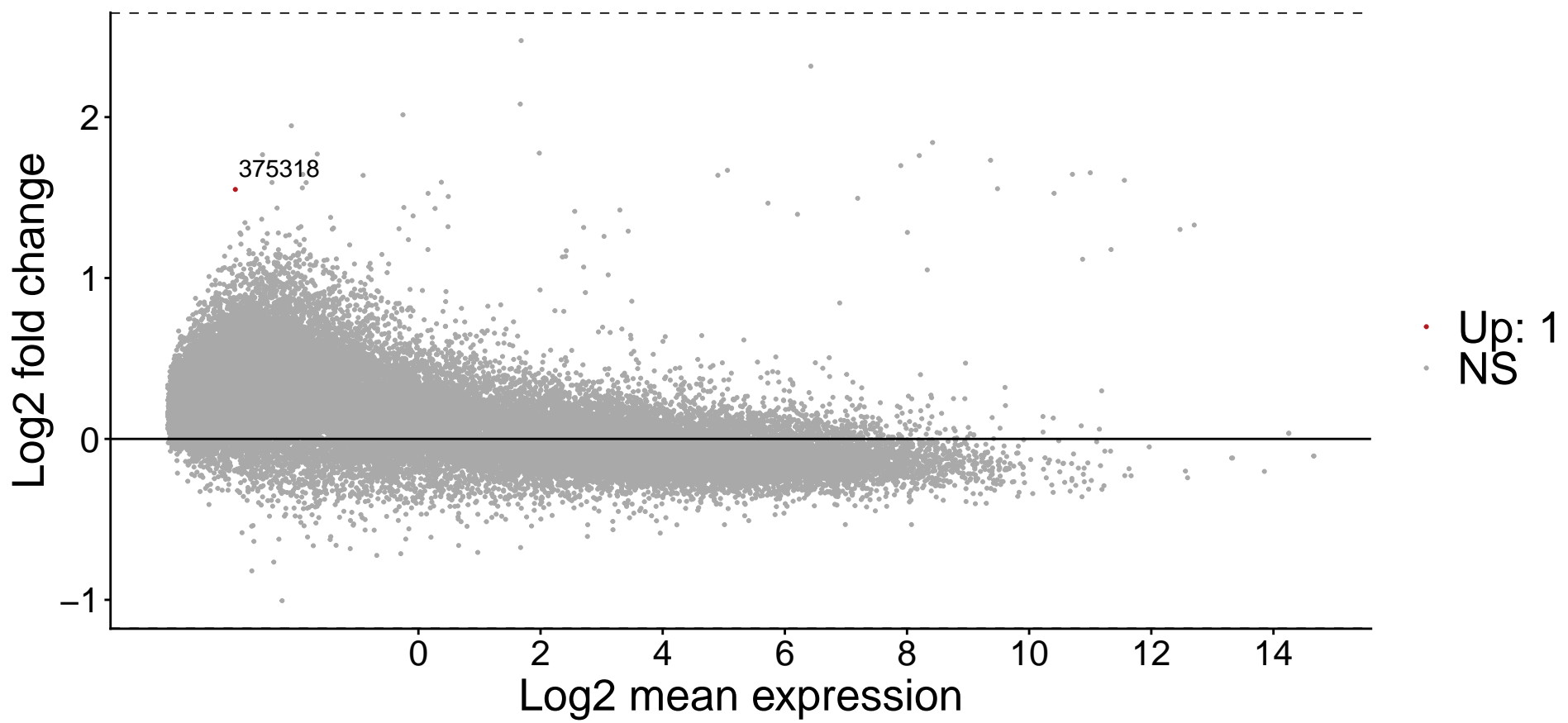**B**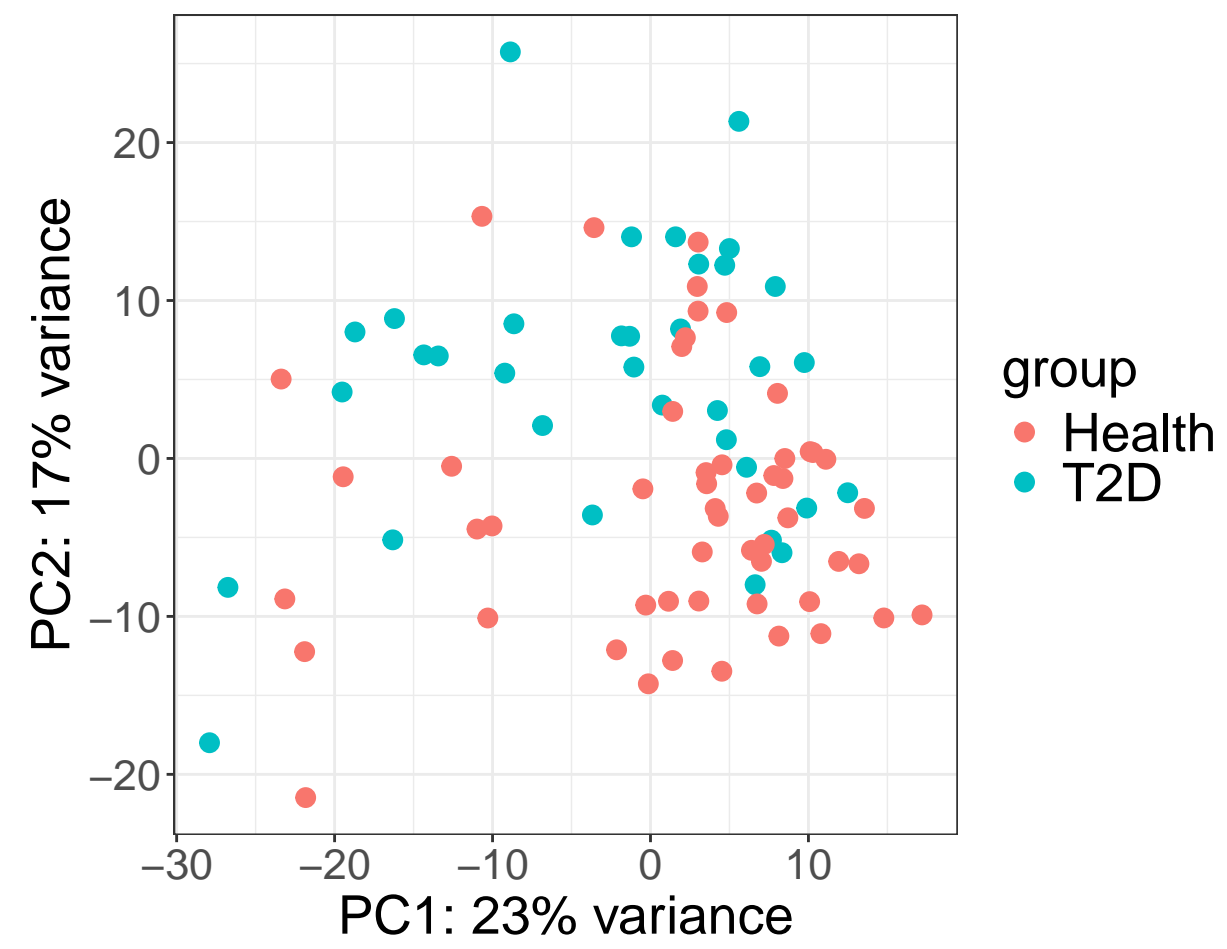**C**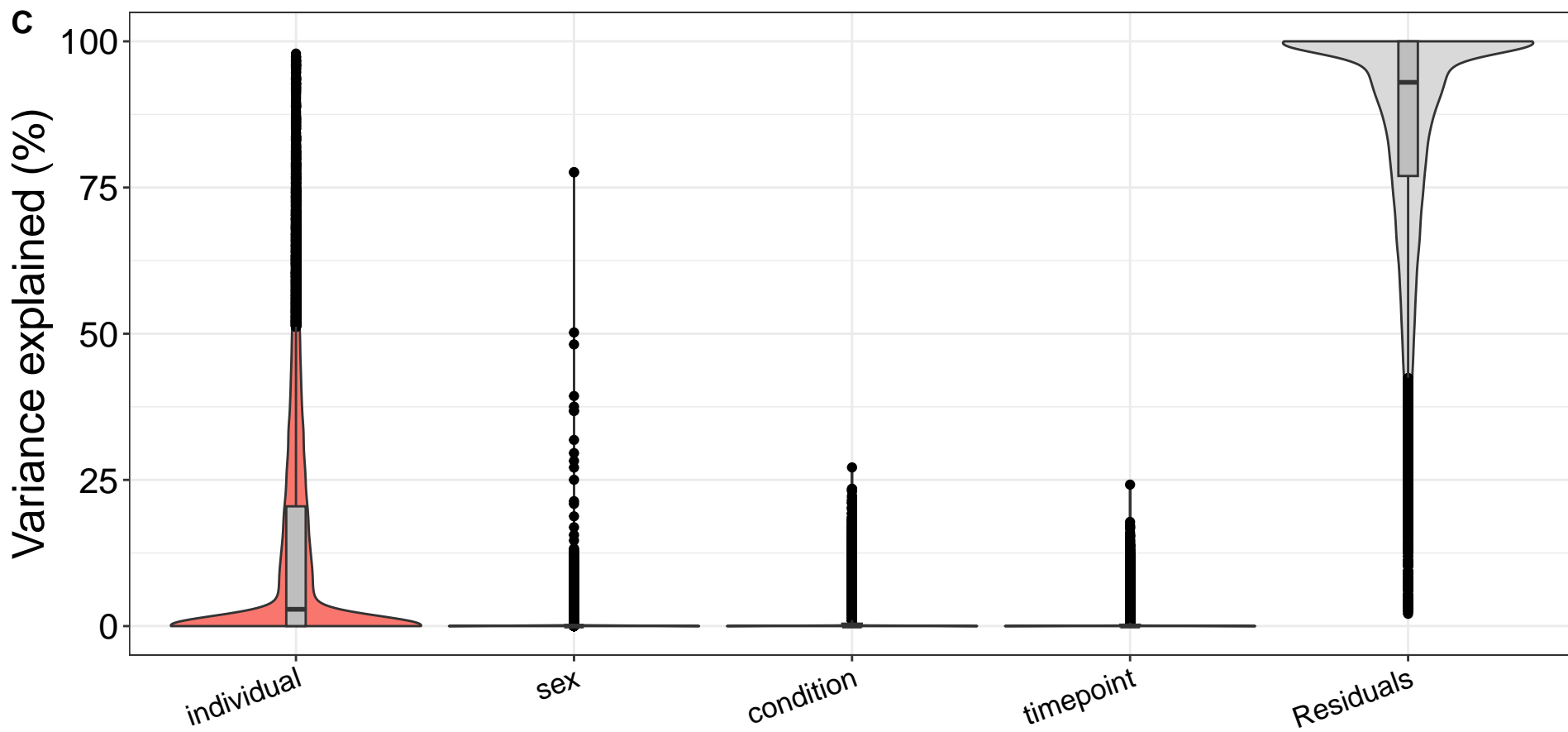

Supplement: Supplementary file 1 [file ijms-26-12046-s001.zip › S4.pdf]

**A** GSE221521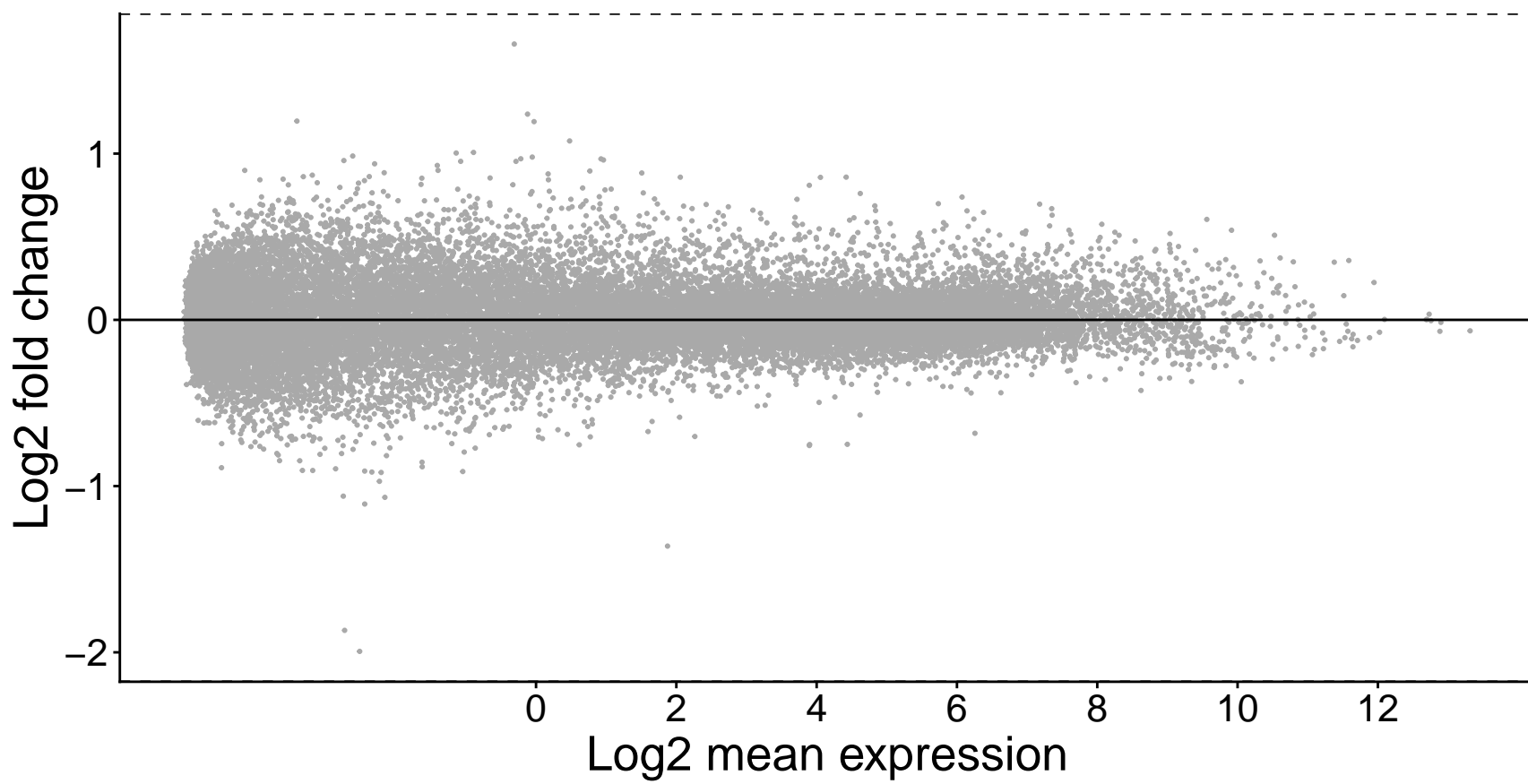

NS

**B**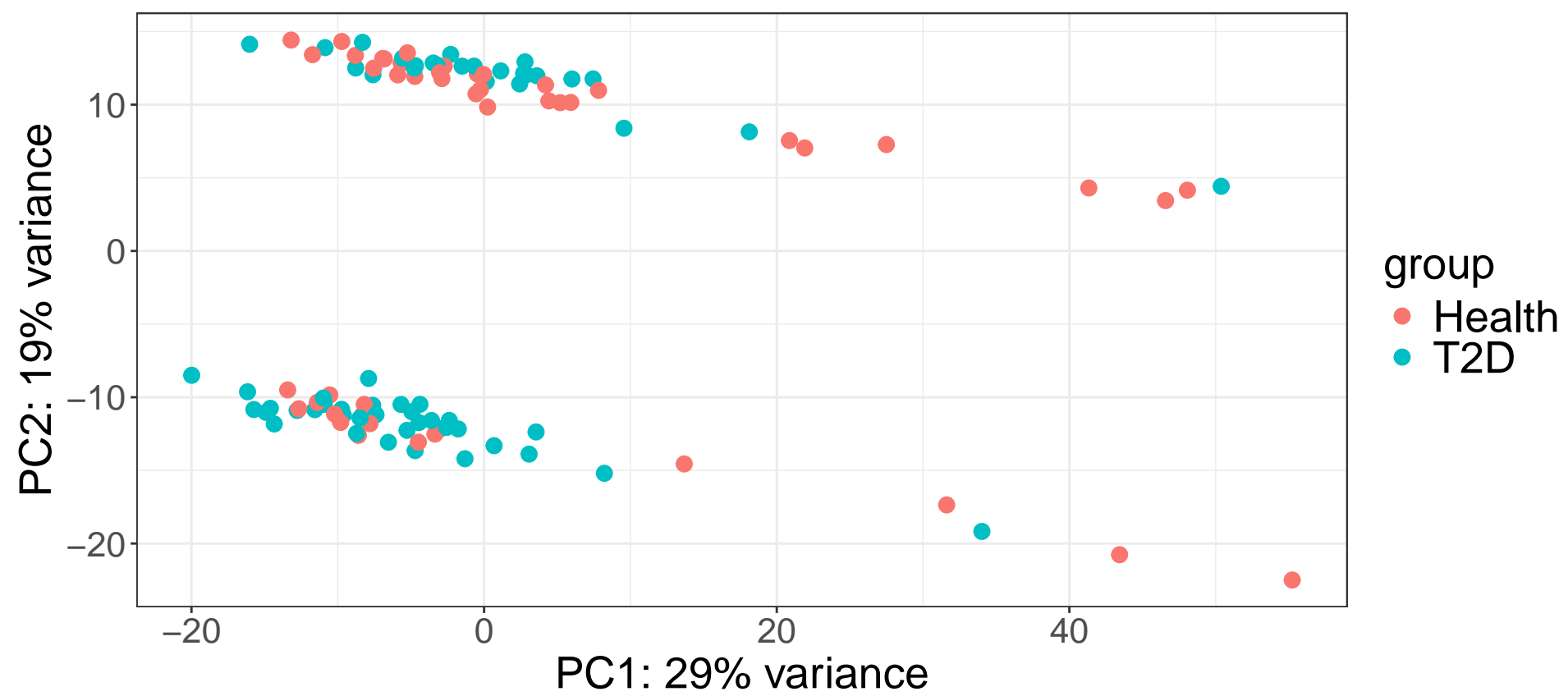**C**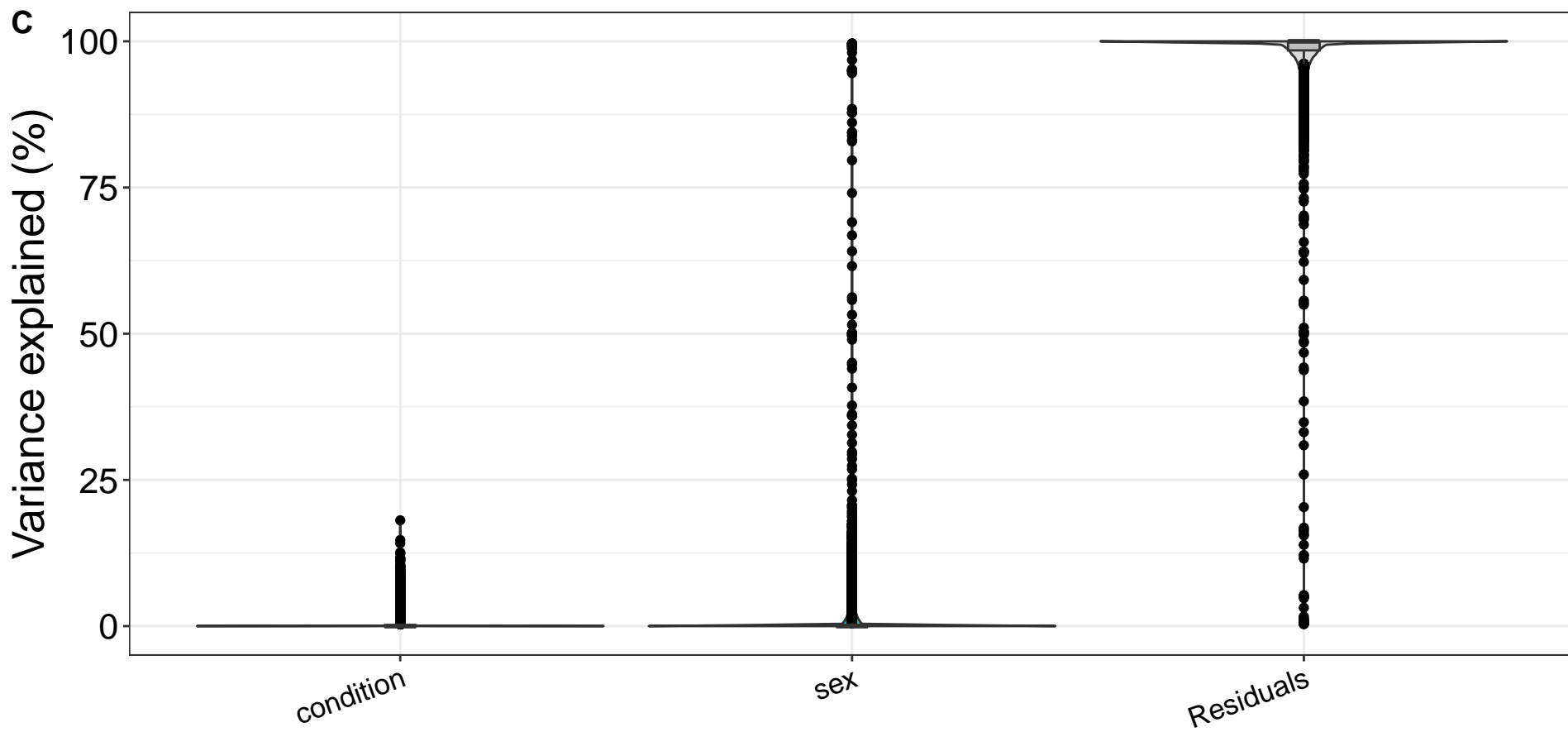

Supplement: Supplementary file 1 [file ijms-26-12046-s001.zip › S5.pdf]

**A** GSE185011

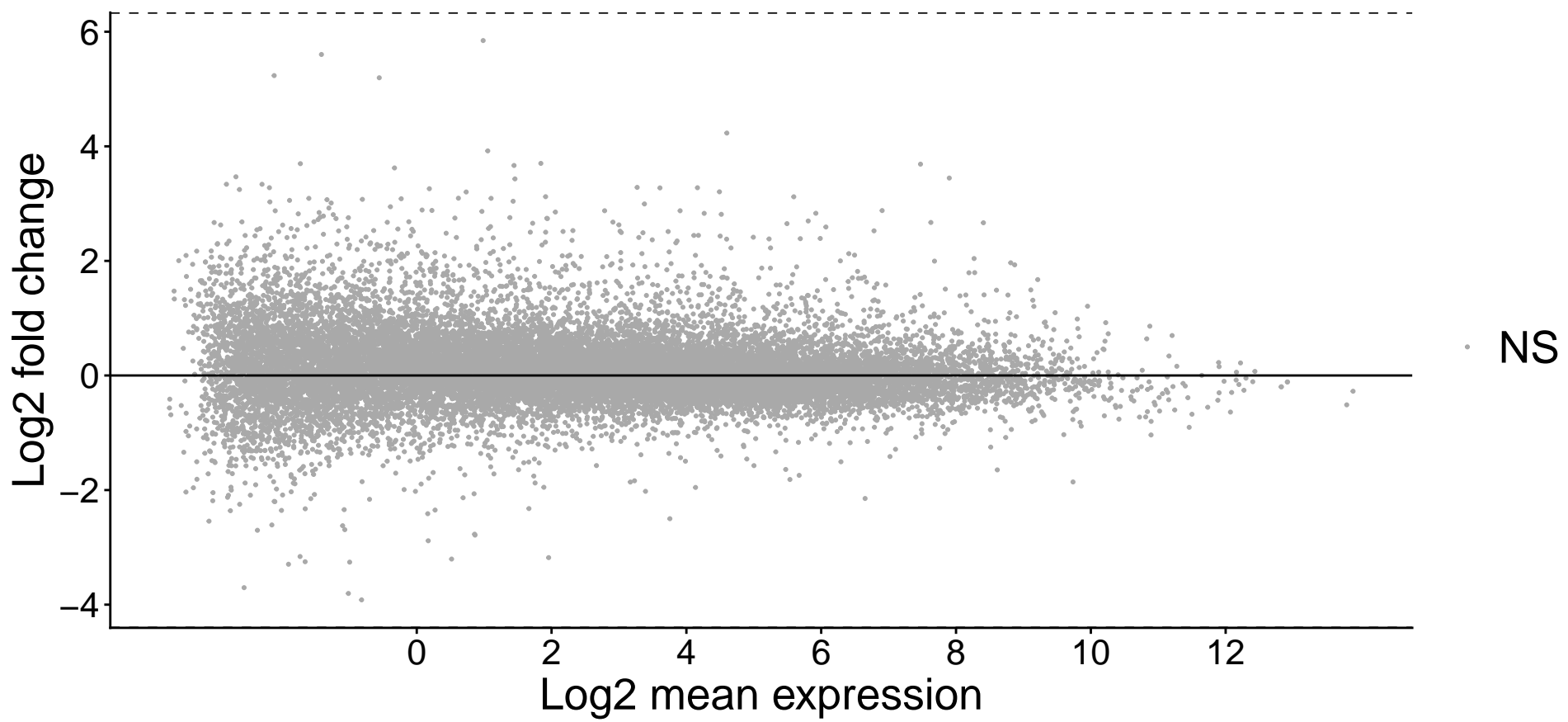

**B**

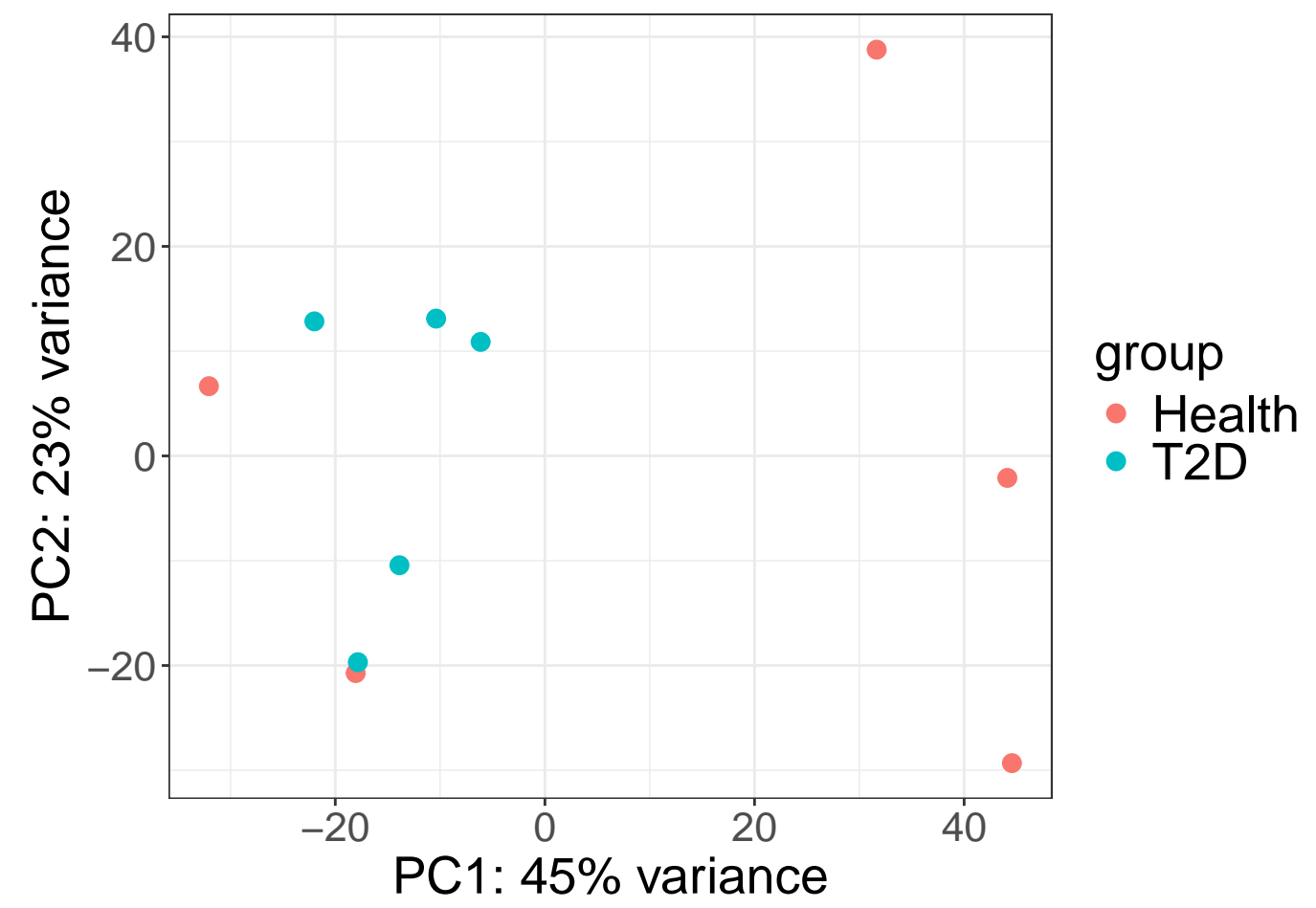

**C**

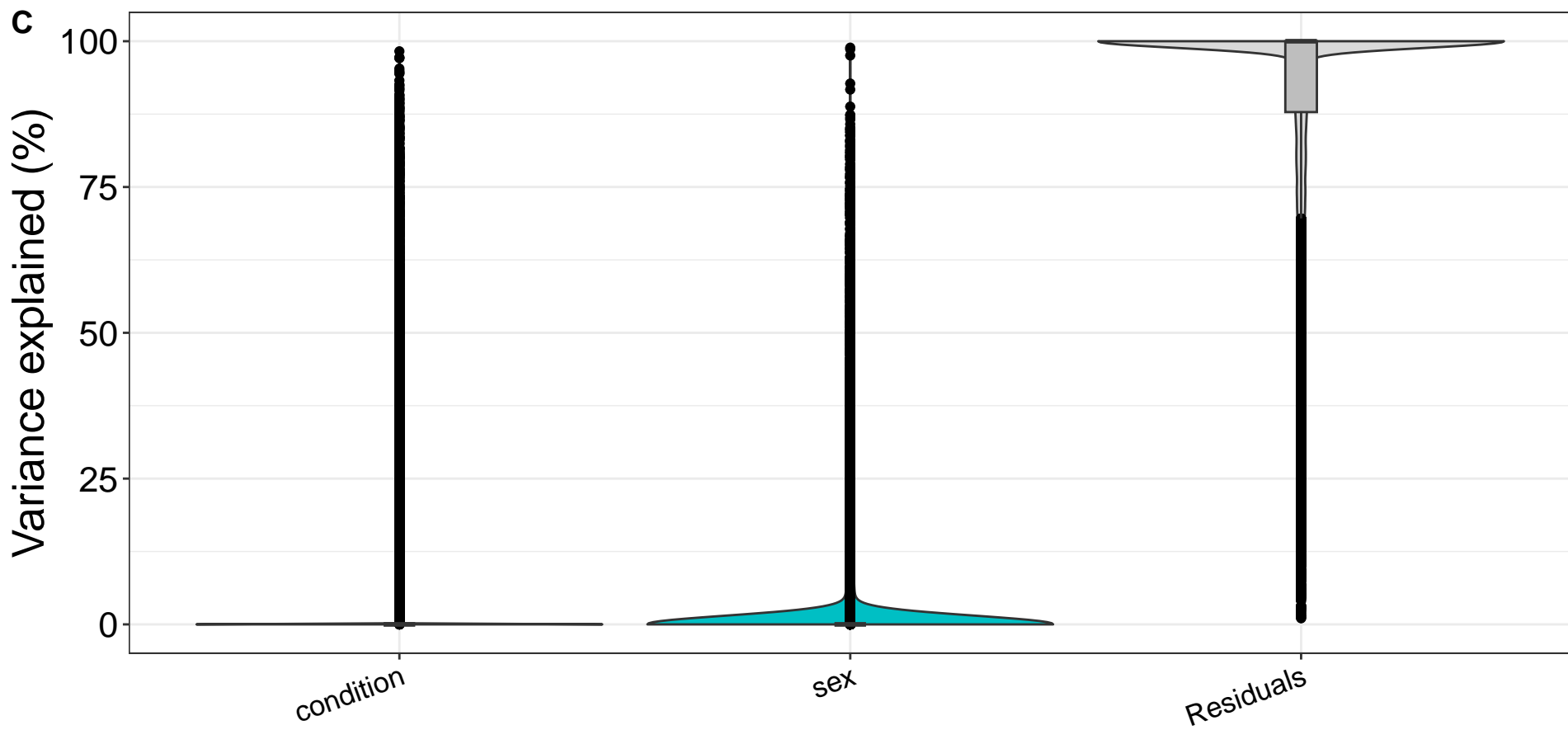

Supplement: Supplementary file 1 [file ijms-26-12046-s001.zip › S6.pdf]

**A** GSE181143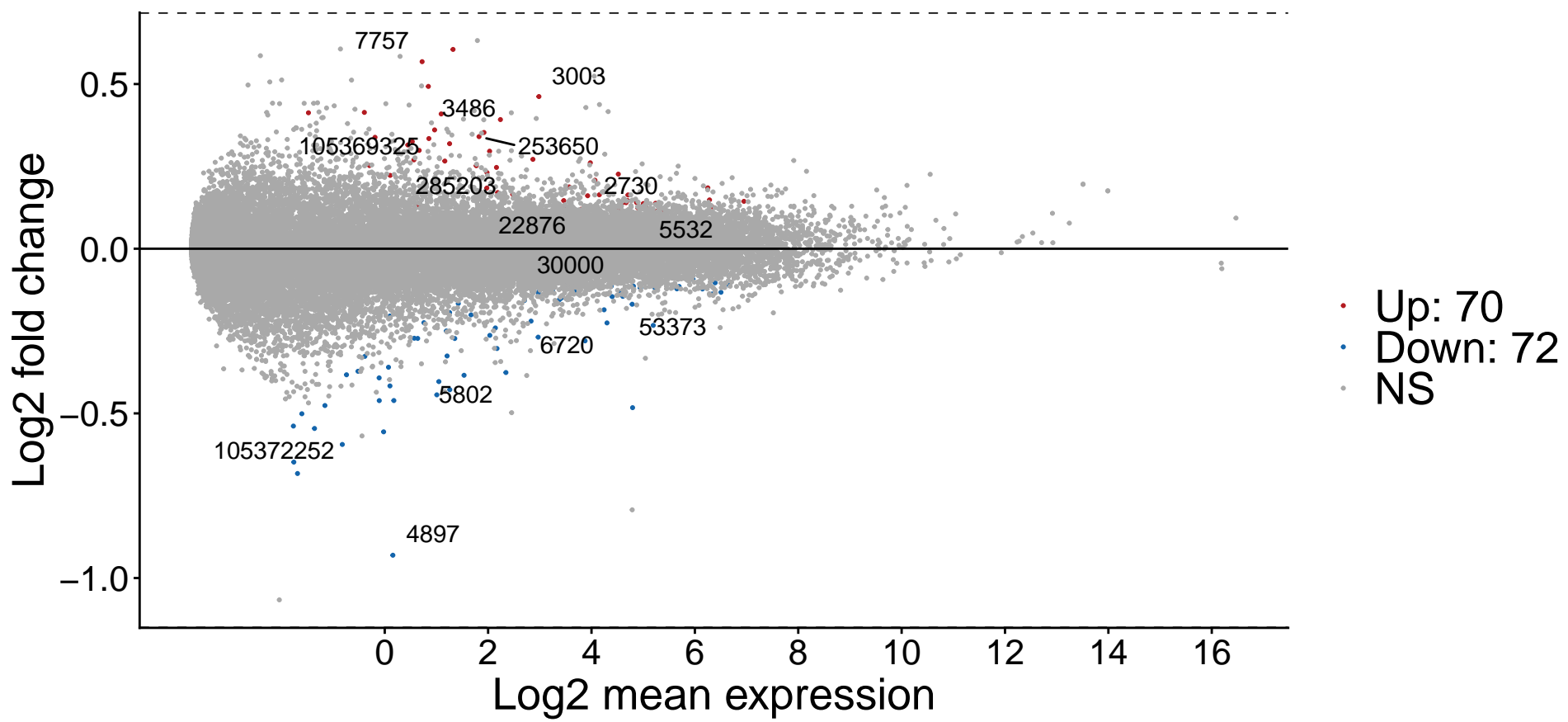**B**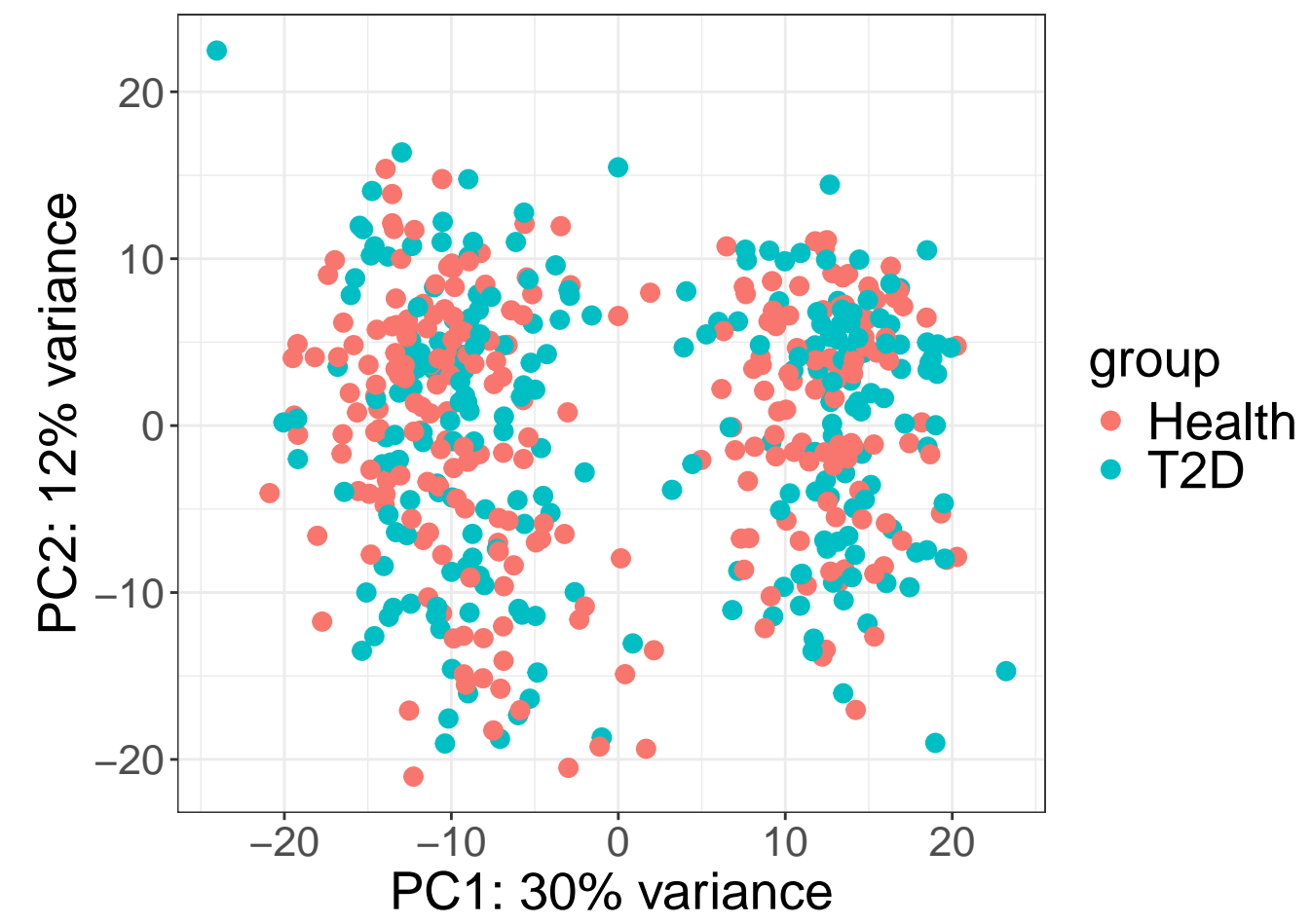**C**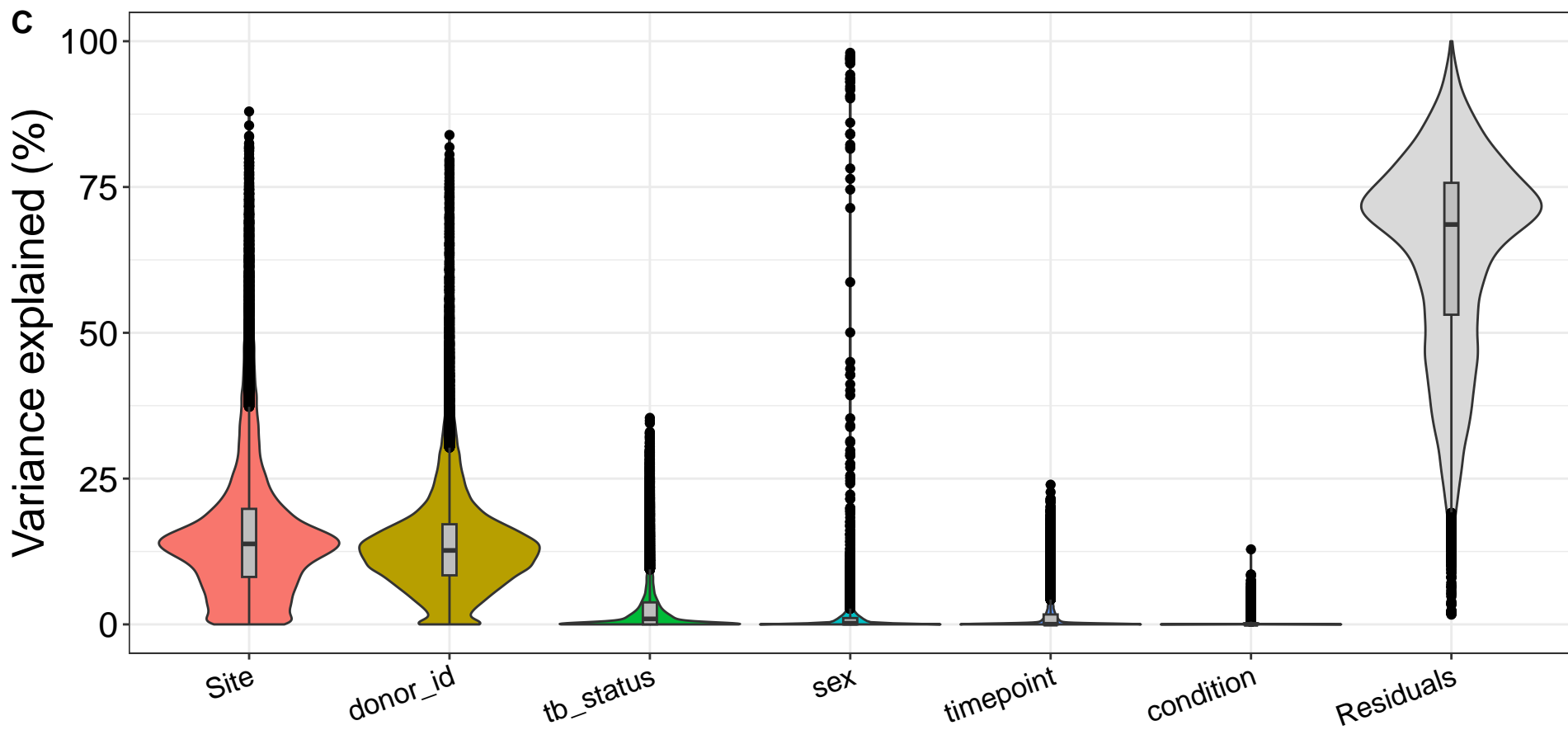

Supplement: Supplementary file 1 [file ijms-26-12046-s001.zip › S7.pdf]

**A** GSE114192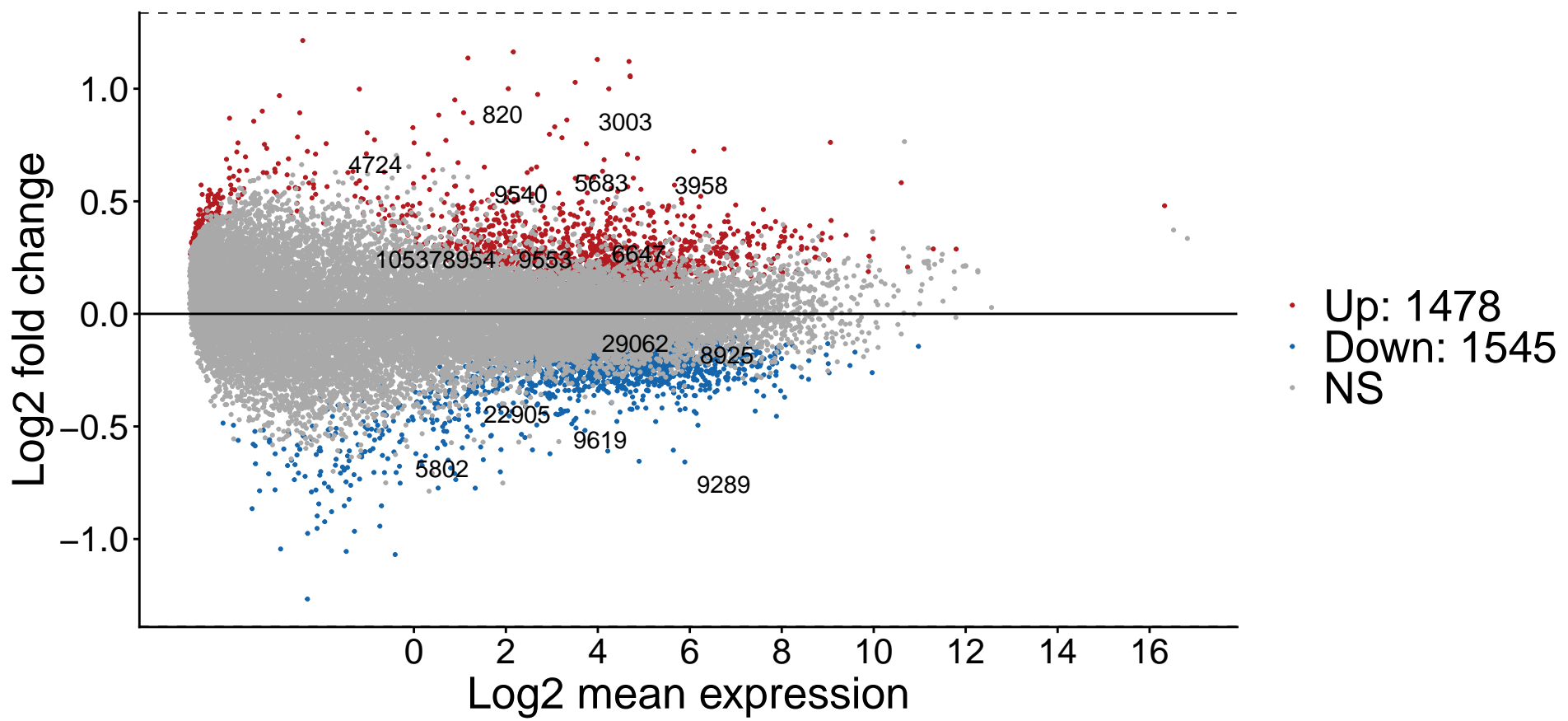**B**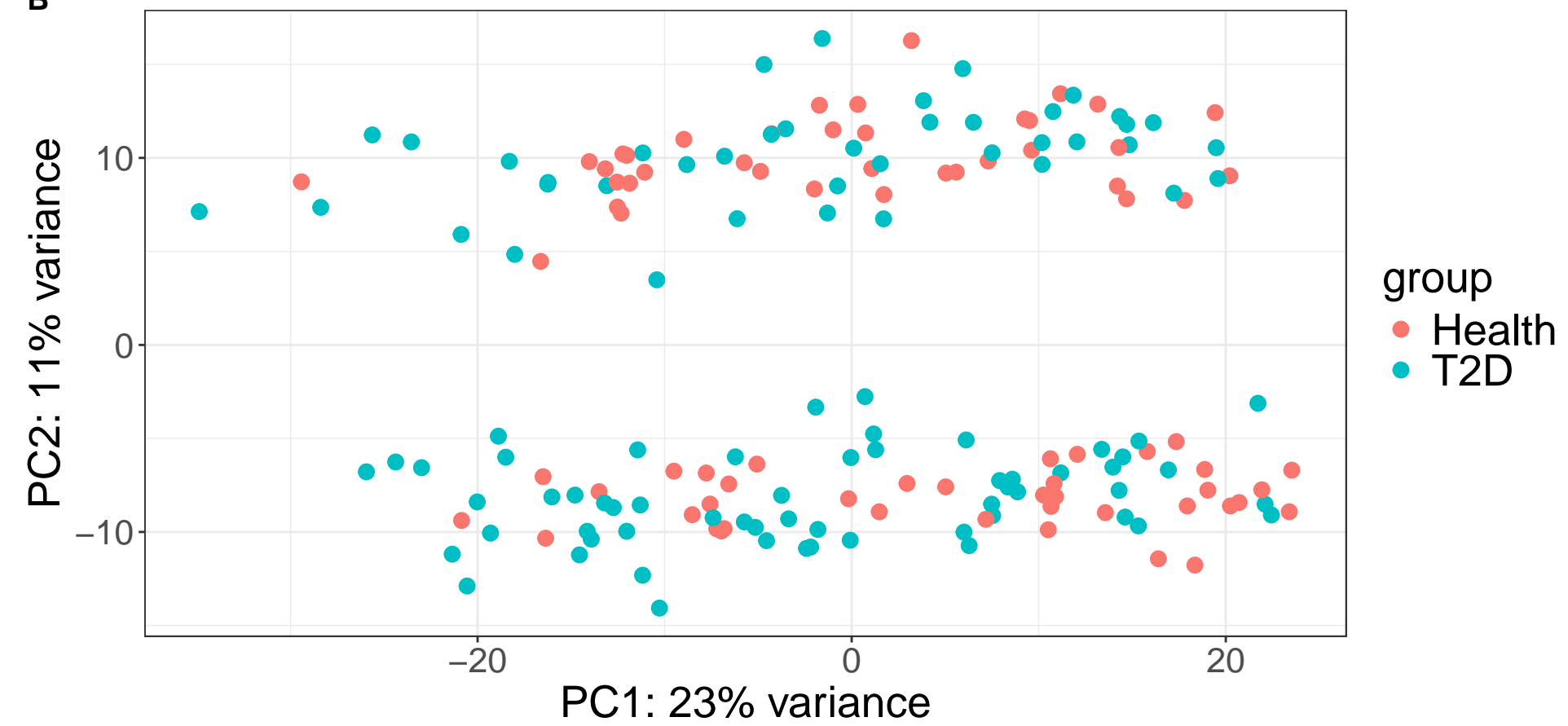**C**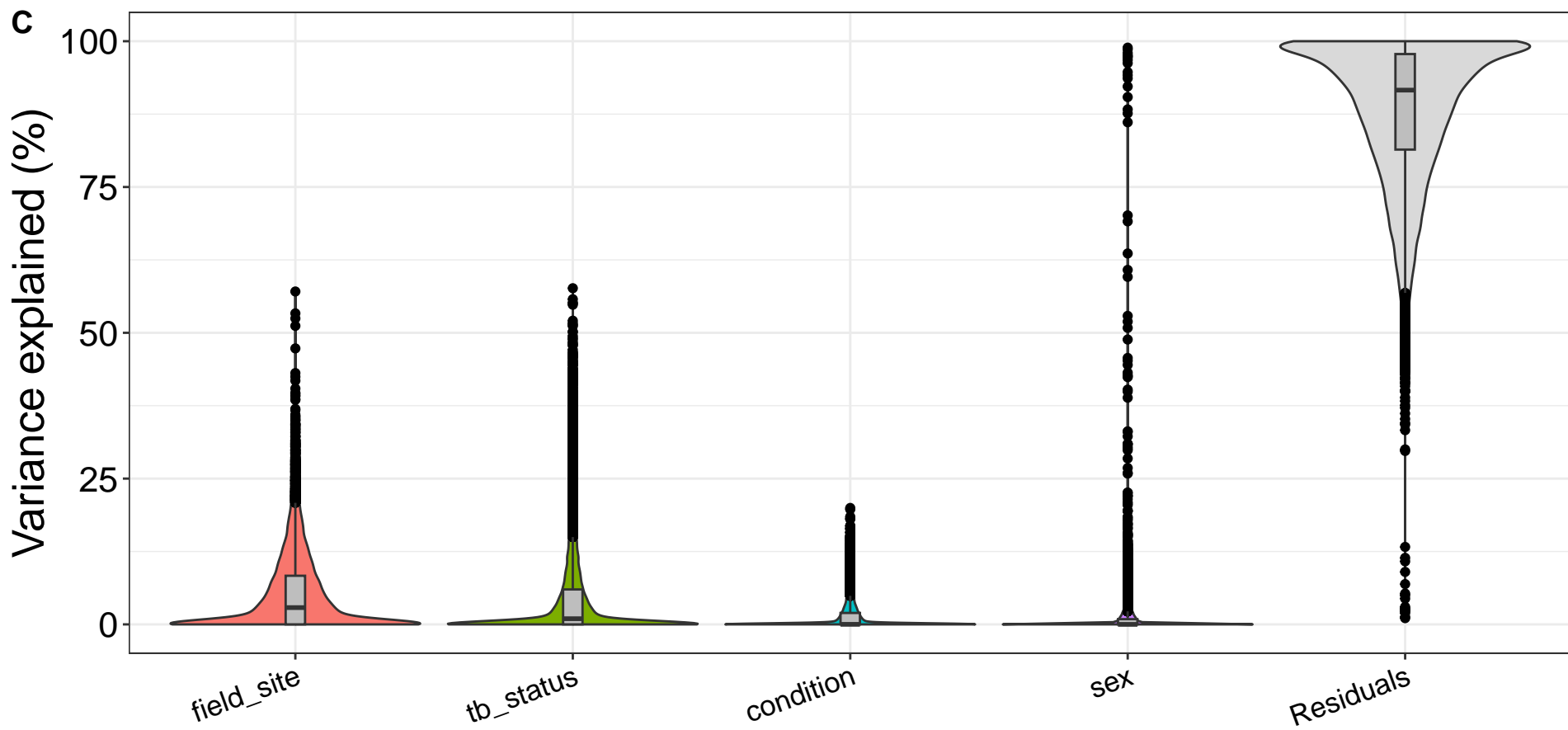

Supplement: Supplementary file 1 [file ijms-26-12046-s001.zip › S8.pdf]
